# Quinacrine-CASIN combination overcomes chemoresistance in human acute lymphoid leukemia

**DOI:** 10.1038/s41467-021-27300-w

**Published:** 2021-11-26

**Authors:** Limei Wu, Srinivas Chatla, Qiqi Lin, Fabliha Ahmed Chowdhury, Werner Geldenhuys, Wei Du

**Affiliations:** 1 Division of Hematology and Oncology, University of Pittsburgh School of Medicine, Pittsburgh, USA; 2 Department of Pharmaceutical Sciences, School of Pharmacy, West Virginia University, Morgantown, WV 26506 USA; 3 Fels Cancer Institute for Personalized Medicine, Lewis Katz School of Medicine at Temple University, Philadelphia, PA 19140 USA; 4 Molecular Pharmacology Graduate Program, University of Pittsburgh School of Medicine, Pittsburgh, USA; 5 UPMC Hillman Cancer Center, Pittsburgh, PA 15213 USA

**Keywords:** Acute lymphocytic leukaemia, Phenotypic screening, Haematopoietic stem cells

## Abstract

Chemoresistance posts a major hurdle for treatment of acute leukemia. There is increasing evidence that prolonged and intensive chemotherapy often fails to eradicate leukemic stem cells, which are protected by the bone marrow niche and can induce relapse. Thus, new therapeutic approaches to overcome chemoresistance are urgently needed. By conducting an ex vivo small molecule screen, here we have identified Quinacrine (QC) as a sensitizer for Cytarabine (AraC) in treating acute lymphoblastic leukemia (ALL). We show that QC enhances AraC-mediated killing of ALL cells, and subsequently abrogates AraC resistance both in vitro and in an ALL-xenograft model. However, while combo AraC+QC treatment prolongs the survival of primary transplanted recipients, the combination exhibits limited efficacy in secondary transplanted recipients, consistent with the survival of niche-protected leukemia stem cells. Introduction of *C*dc42 *A*ctivity *S*pecific *In*hibitor, CASIN, enhances the eradication of ALL leukemia stem cells by AraC+QC and prolongs the survival of both primary and secondary transplanted recipients without affecting normal long-term human hematopoiesis. Together, our findings identify a small-molecule regimen that sensitizes AraC-mediated leukemia eradication and provide a potential therapeutic approach for better ALL treatment.

## Abstract (web summary)

Chemoresistance and relapse are main limitations in the treatment of acute lymphoblastic leukemia (ALL). Here, the authors identify Quinacrine (QC) as a sensitizer for Cytarabine (AraC) and establish a QC-CASIN regimen to improve leukemia eradication in ALL.

## Introduction

Acute lymphoid leukemia (ALL) is the most common pediatric cancer, with a peak incidence at 2–5 years of age^1^. Despite cure rates approaching 90% due to better management and improved therapies, resistance to treatments and disease relapse remain a significant clinical problem^2,3^. In fact, more than half of adults and about 20% of children who are diagnosed with ALL and achieve remission with combination cytotoxic chemotherapy will eventually relapse^4,5^. Identification of the genes and biological pathways responsible for chemoresistance is therefore crucial for the design of novel therapeutic approaches aiming to improve patient survival^6^.

Nucleoside analogs represent a group of cytotoxic antimetabolites in the treatment of hematological malignancies, solid tumors, and viral infections^7–10^. Their cytotoxic activity is based on the interference with cellular targets involved in the metabolism of physiological nucleosides and DNA synthesis^11^. Cytarabine (1-β-D-arabinofuranosylcytosine; AraC), a deoxycytidine analog, which exerts its cytotoxic effects by disrupting normal DNA synthesis through direct incorporation in extending DNA strands^12^, is used in the upfront treatment of ALL and has become one of the most important anti-leukemia drugs currently available for the treatment of acute myeloid leukemia (AML)^13,14^, relapsed and refractory ALL^15–17^, and large cell lymphoma^18^. However, prolonged in vitro and in vivo, treatment with AraC has resulted in the emergence of cells with diminished sensitivity to the drug and ultimately contributed to treatment failures^14,19–21^. Therefore, novel therapeutic approaches that can improve AraC efficacy and reduce the risk of relapse are urgently needed.

Quinacrine (QC), a derivative of 9-aminoacridine and an anti-inflammatory drug used extensively to treat malaria and rheumatoid arthritis, has been identified as a potential anti-cancer agent. For example, QC has been shown to up-regulate p53 and down-regulate NF-κB, thereby causing a decrease in the survival of renal cell carcinoma cells^22,23^. QC enhances temozolomide cytotoxicity in temozolomide-sensitive and resistant glioblastoma cells^24^. Another study suggests that QC promotes autophagic cell death and chemosensitivity, leading to inhibition of tumor growth in ovarian cancer^25^. Although the scattered in vitro studies suggest a potential of QC in treating AML in vitro^26^, whether QC has an effect in other leukemia, especially in refractory ALL is not known.

The Rho family small GTPase Cdc42 acts as an intracellular signal transducer in response to a variety of extracellular stimuli^27,28^. A recent study using a conditional knockout mouse model has shed light on the mechanisms of HSC regulation related to bone marrow (BM) niche localization^29,30^ and suggests that Cdc42 is uniquely required for hematopoietic stem cell (HSC) retention and maintenance in the BM. More recently, we rationally identified a *C*dc42 *A*ctivity *S*pecific *In*hibitor (CASIN) that inhibits the activity of Cdc42 specifically and transiently to induce hematopoietic stem/progenitor cell (HSPC) egress from the BM by suppressing actin polymerization, cell polarity, adhesion, and directional migration^31^. We showed that mimicking the effect of *Cdc42* gene targeting, CASIN induces HSC mobilization from mouse BM and renders BM niche accessible for murine and human donor HSC engraftment without myeloablative pre-conditioning^32^.

In the current study, we identified QC as a potential sensitizer for AraC in ALL treatment through an ex vivo small molecule screen. We further show that QC enhances the cytotoxicity of AraC in resistant ALL cells in ex vivo culture and delays ALL development in a patient-derived xenograft model. The introduction of CASIN enhances the eradication of ALL leukemia stem cells (LSCs) by AraC + QC and prolongs the survival of both primary and secondary recipients by mobilizing niche-protected leukemia-initiating cells (LICs) from BM to peripheral blood (PB) without affecting normal human hematopoiesis. We propose that CASIN-QC combination is a promising strategy to overcome AraC resistance in ALL treatments.

## Results

### Identification of QC, a chemosensitizing agent that overcomes AraC resistance in ALL

In an attempt to search for new chemosensitizing agents that are effective and less toxic in refractory (ALL) treatment, we performed an ex vivo screen of the LOPAC^1280^ Library^33–35^, a collection of 1280 pharmacologically active compounds using our newly established AraC-resistant Molt4-Luc2 cell line, an ALL cell line stably transfected with firefly luciferase gene (Luc2; Supplementary Fig. 1A)^36^.

By employing Synergy H1 luminescent assay, we identified 45 potential candidates out of initial screening (Supplementary Fig. 1B, Supplementary Table 1). Further validation indicated that 18 out of 45 exhibited a significant inhibitory effect on AraC-resistant Molt4-Luc2 cells (Supplementary Fig. 1C; Supplementary Table 1). Among several candidates that showed a high inhibitory effect on AraC-resistant leukemic cells, QC was among the top 2 hits that inhibited AraC-resistant cell growth. We decided to focus on QC because the drug has been shown to inhibit leukemic cell growth in several leukemia models^23,26^ and because we found the drug consistently effective in both leukemic cell lines and primary samples (see below).

**Fig. 1:**
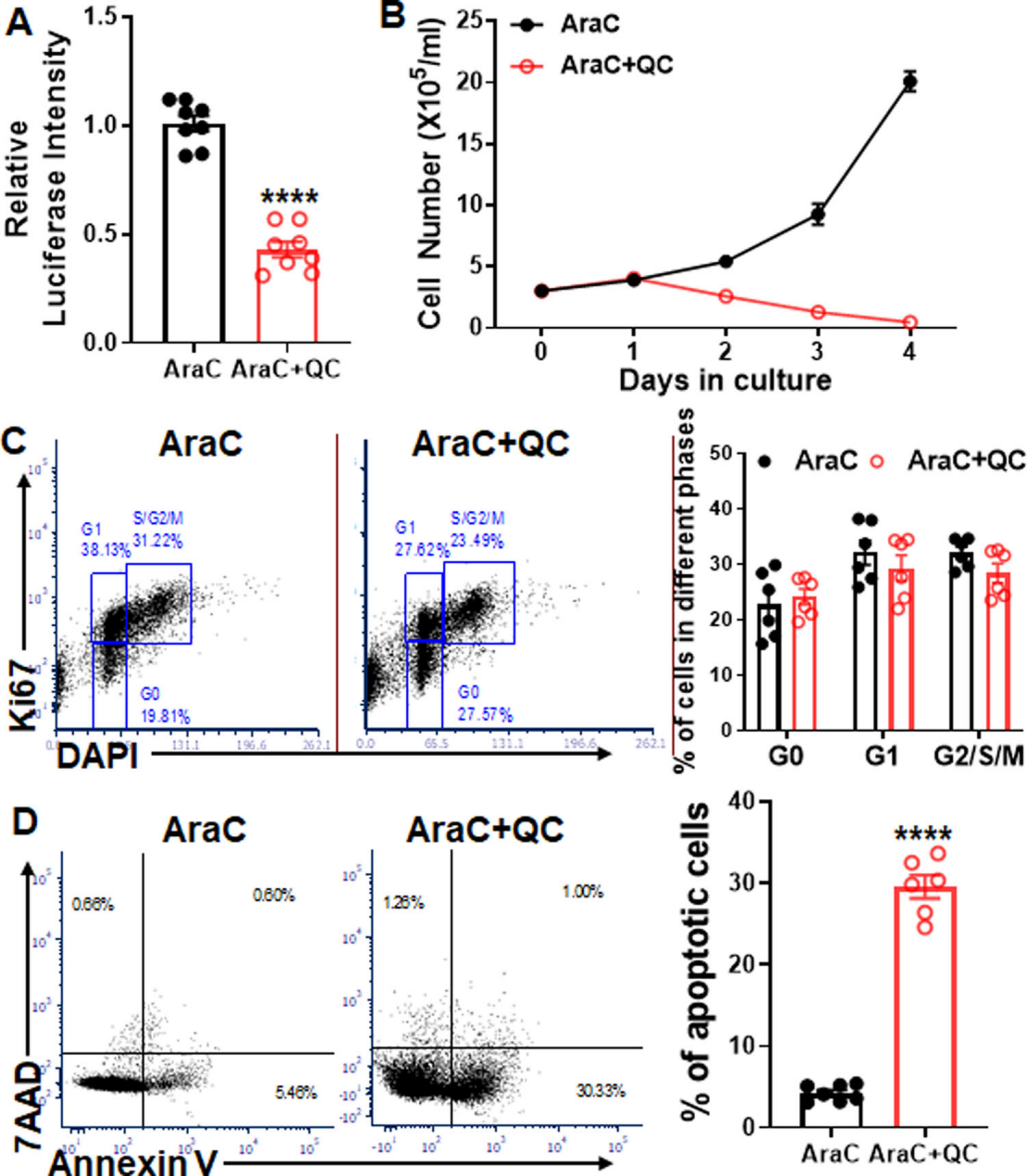
QC enhances the cytotoxicity of AraC in resistant Molt4-Luc2 ALL cells in vitro. **A** QC inhibits AraC-resistant Molt4-Luc2 cell growth. Totally, 50,000 AraC-resistant Molt4-Luc2 cells were seeded in each well of 96-well plates and cultured in the presence of AraC (2 μM). A 100 nM of QC was then added to each well. Luciferase intensity of each well was measured after 24 h culture. Results are mean ± standard error of the mean (SEM) of three independent experiments (*n* = 8). **B** QC inhibits the growth of AraC-resistant Molt4-Luc2 cells. AraC-resistant Molt4-Luc2 cells were seeded in 6-well plates for culture with AraC (2 μM) in the presence or absence of QC (100 nM). Absolute cell numbers were enumerated and plotted at the indicated time points. Results are mean ± SEM of two independent experiments (*n* = 6). **C** QC does not affect the proliferation of AraC-resistant cells. AraC-resistant Molt4-Luc2 cells were treated with QC followed by flow cytometry analysis at 48 h for Ki67 and DAPI. Representative flow plots and quantification are shown. Results are mean ± SEM of three independent experiments (*n* = 6). **D** QC induces apoptosis of AraC-resistant Molt4-Luc2 cells in the presence of AraC. AraC-resistant cells were cultured in the presence of AraC (2 μM) with or without QC (100 nM) for 24 h followed by flow cytometry analysis for Annexin V and 7AAD. Representative flow plots and quantification are shown. Results are mean ± SEM of three independent experiments (AraC, 7; AraC+QC: 6). See also Supplementary Fig. 1. Statistics were performed in the indicated groups: two-tailed, paired *t* test (parametric); *p* values are indicated in Source Data files (***p < 0.001; ****p < 0.0001).

QC is a derivative of 9-aminoacridine and an anti-inflammatory drug used extensively to treat malaria and rheumatoid arthritis and has been identified as a potential anticancer agent^22,24,25^. We first confirmed that QC significantly reduced the luciferase intensity of AraC-resistant Molt4-Luc2 cells after 24 h culture by luminescence assay (Fig. 1A). Concomitantly, the cell growth was substantially inhibited in AraC + QC cultured cells compared to those cultured with AraC alone (Fig. 1B, Supplementary Fig. 2), suggesting an additive inhibitory effect of QC on AraC-resistant Molt4-Luc2 cells. Flow cytometry analysis showed that while QC did not affect the cell cycle status of AraC-resistant cells (Fig. 1C), QC treatment significantly induced apoptosis (Fig. 1D), suggesting a potential of QC in sensitizing AraC cytotoxicity on ALL cells.

### QC enhances the cytotoxicity of AraC in ALL cell lines in vivo

**Fig. 2:**
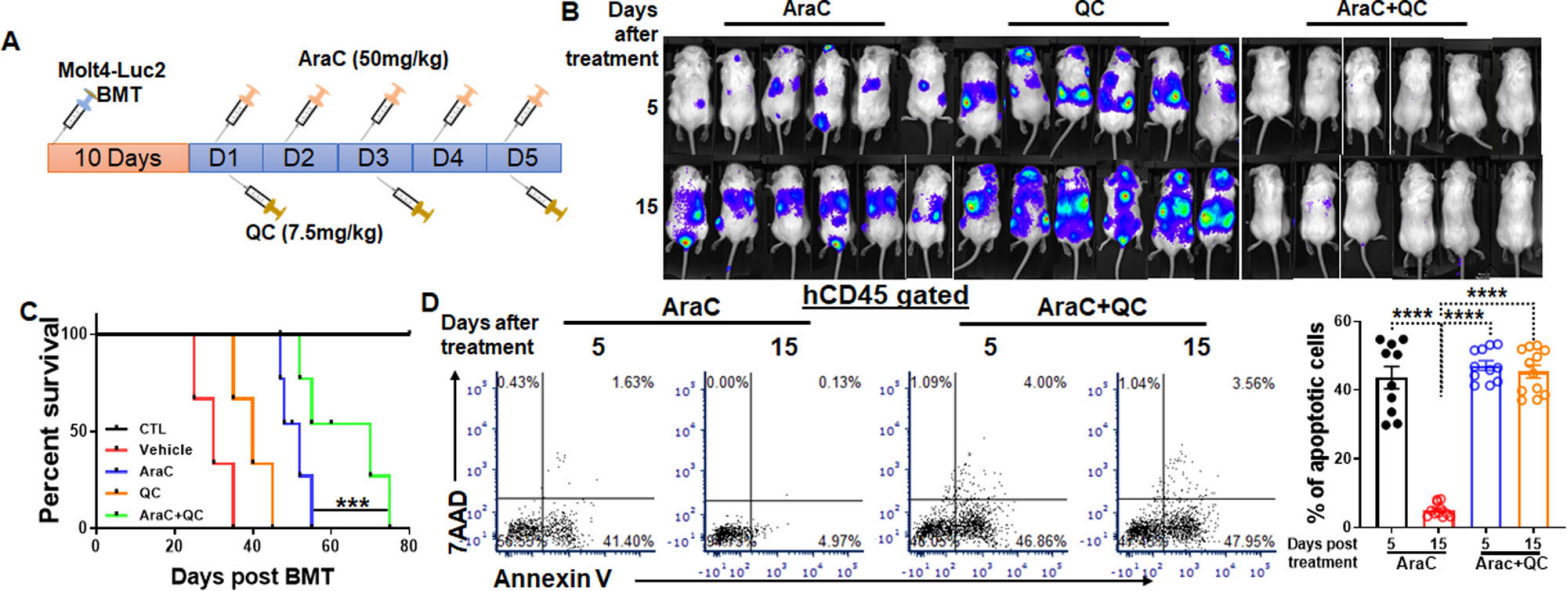
QC delays ALL development but fails to prevent ALL relapse in vivo. **A** Schematic presentation of experimental design. One million Molt4-Luc2 cells were transplanted into each sublethally irradiated NSGS recipient. Ten days post bone marrow transplantation (BMT), vehicle, AraC, QC, or AraC + QC were administered daily to the recipients for consecutive 5 days followed by IVIS imaging analysis at the indicated time points. **B** QC enhances short-term clearance of ALL cells by AraC but failed to prevent ALL relapse in xenografted mice. The distribution (luciferase intensity) of Molt4-Luc2 cells in the mice described in (**A**) were determined by visualizing luciferase using the IVIS system at the indicated time points (*n* = 6/group). **C** QC administration improves the survival of primary recipients transplanted with ALL cells. Survival of the recipients was monitored and plotted by the Kaplan–Meier method (Ctr, *n* = 8; Vehicle, *n* = 9; AraC, *n* = 12; QC, *n* = 9; AraC+QC, *n* = 12). Median survival: Vehicle (30 days); AraC (52 days); QC (40 days); and AraC + QC (70 days). **D** QC enhances the apoptotic effect of AraC on ALL cells in vivo. Human donor-derived cells (hCD45^+^) from BM of the recipients described in (**A**) were aspirated and subjected to Flow cytometry analysis for apoptosis at different time points. Representative flow plots (Left) and quantification (Right) are shown. Results are means ± SEM of three independent experiments (AraC, 5 days: *n* = 10; 15 days: *n* = 11; AraC + QC, 5 days, *n* = 11; 15 days, *n* = 12). See also Supplementary Fig. 3. Statistics were performed in the indicated groups: two-tailed, paired *t* test (parametric); *p* values are indicated in Source Data files (**p < 0.01; ***p < 0.001; ****p < 0.0001).

To determine whether QC could enhance the efficacy of chemotherapy in vivo, we employed our previously established human ALL-NSGS xenograft model^37^, which consistently mimics the human ALL remission and relapse in vivo (Supplementary Fig. 3). Totally, 10 days posttransplant, a regimen consisting of QC and AraC was applied to 2 xenotransplant models: Molt4-Luc2 and primary ALL patient cells. We first examined the effect of combined QC and AraC in humanized NSGS mice transplanted with Molt4-Luc2 cells (Fig. 2A) and found that combination of QC and AraC markedly reduced leukemia burden in the recipients compared to AraC or QC alone, as determined by the Xenogen IVIS imaging system (Fig. 2B). It is noteworthy that leukemia cells were suppressed by AraC alone but came back 10–15 days post-treatment in this xenotransplant model (Fig. 2B and Supplementary Fig. 3D)^38^. Significantly, combined AraC + QC treatment prolonged the life span of Molt4-Luc2 cell-xenografted mice compared to those treated with single-agent (Fig. 2C). Consistently, xenografted leukemic cells from AraC + QC-treated mice at 15 days post treatment exhibited profoundly increased apoptosis compared to those of the AraC alone group (Fig. 2D). These results indicate that combined AraC + QC treatment prolongs the survival of the primary recipients transplanted with ALL cells.

### QC increases AraC response on primary ALL cells in vitro but has no effect on L-LTC-ICs

**Fig. 3:**
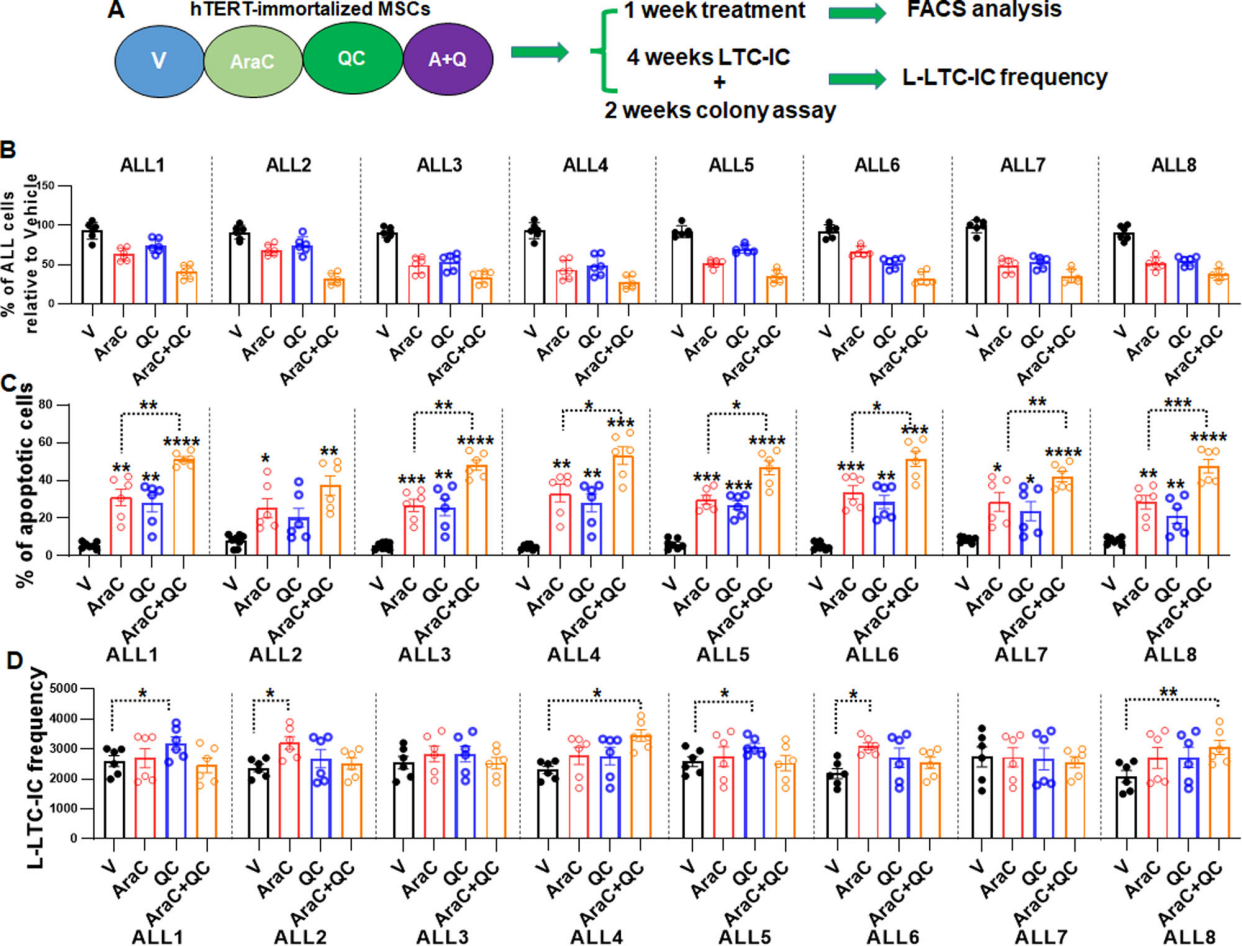
QC enhances AraC cytotoxicity in primary ALL cells in vitro but has no effect on L-LTC-ICs. **A** Schematic presentation of short-term culture (STC) and long-term culture (LTC) of primary ALL cells. **B** QC enhances AraC cytotoxicity in primary ALL cells in culture. Percentage of ALL cell survival after treatment was determined using counting beads and normalized to the Vehicle control. Results are means ± SEM of two independent experiments (*n* = 6). **C** QC increases the apoptotic effect of AraC on ALL cells in vitro. Apoptosis of eight ALL samples 1 week after treatment was measured by Flow Cytometry using Annexin V/7AAD staining. Results are means ± SEM of three independent experiments (Vehicle: ALL1, 5, 7, 8, *n* = 7; ALL2, 3, 4, 6, *n* = 8; AraC, QC, AraC + QC, *n* = 6 for all groups). **D** QC does not increase the effect of AraC on ALL long-term culture-initiating cells (LTC-ICs). Eight ALL samples were subjected to 1 week of treatments with the indicated regimens, 4 weeks of LTC in a limiting dilution assay (LTC-LDA), and 2 weeks of culture in methylcellulose, followed by determination of the L-LTC-IC frequencies. V, Vehicle; A, AraC; QC: Quinacrine; A + QC, AraC + Quinacrine. Results are means ± SEM of three independent experiments (*n* = 6). Statistics were performed in the indicated groups: two-tailed, paired *t* test (parametric); *p* values are indicated in Source Data files (*p < 0.05; **p < 0.01; ***p < 0.001; ****p < 0.0001).

Next, we examined whether the combination of AraC + QC was also beneficial in primary ALL cells. We tested eight ALL samples with different clinical characteristics (Supplementary Table 2). Primary ALL cells were cultured on hTERT-immortalized MSCs^39,40^ and treated for one week with AraC (500 nM) in the presence or absence of QC (100 nM) (Fig. 3A). We observed a significant decrease in survival of the ALL cells treated with the combo AraC + QC regimen compared to those treated with either single agent (Fig. 3B). Consistently, higher apoptotic ALL cells were observed in the AraC + QC group than single-agent groups (Fig. 3C).

To examine whether the combo AraC + QC treatment was affecting not only the leukemic blasts but also the LSC-enriched long-term culture-initiating cells (L-LTC-IC), we collected the surviving cells after one week of treatment and seeded them at limited dilution in an LTC assay^39,40^. Limited dilution assay (LDA) showed that L-LTC-IC frequency remained similar, or even increased in some patient samples treated with combo AraC+QC or single agent compared to vehicle controls (Fig. 3D), indicating that these chemotherapy regimens do not have a significant effect on the LSC-enriched L-LTC-ICs. Taken together, these results indicate that while QC enhances the killing of proliferating primary ALL cells by AraC, the combo AraC + QC treatment does not promote the elimination of the LSC-enriched L-LTC-ICs.

### QC enhances the cytotoxicity of AraC in primary ALL cells but fails to prolong the survival of secondary recipients

**Fig. 4:**
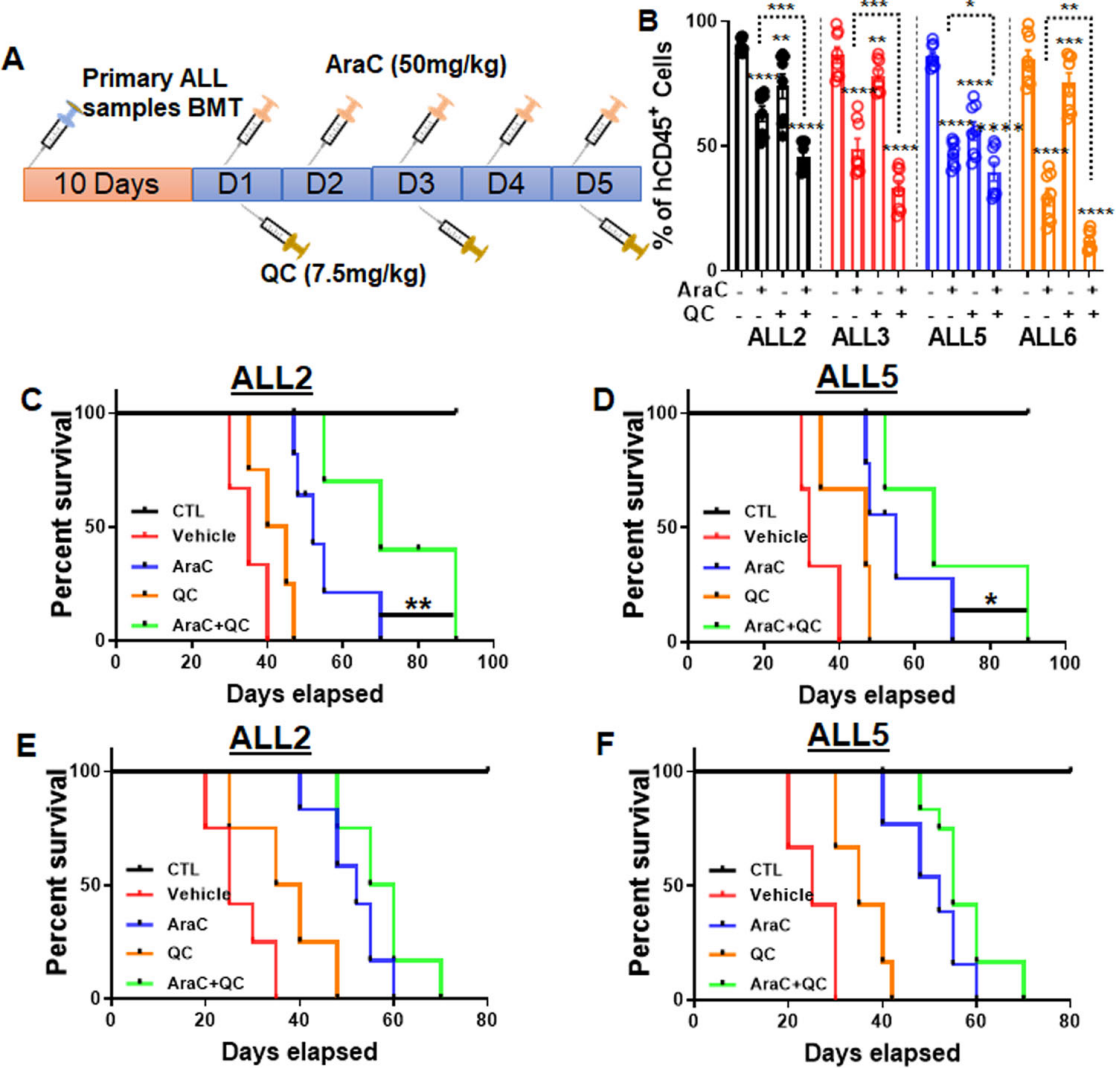
QC enhances the cytotoxicity of AraC in primary ALL cells but fails to prolong the survival of secondary recipients. **A** Schematic presentation of experimental design. **B** QC reduces leukemia burden in primary recipients. Percentage of human cells in the bone marrow of mice transplanted with four ALL patient samples were determined by flow cytometry 1-month post-transplant (ALL2: V, *n* = 10; *n* = 8 for all other groups). **C**, **D** QC prolongs the survival of primary recipients. Survival of recipients transplanted with ALL2 (**C**) or ALL5 (**D**) cells were monitored and plotted by the Kaplan–Meier method (ALL2: Ctr, *n* = 8; V, *n* = 9; AraC; *n* = 10; QC, *n* = 8; AraC + QC, *n* = 9; ALL5: Ctr, *n* = 8; V, *n* = 9; AraC, *n* = 8; QC, *n* = 12; AraC + QC, *n* = 12). Median survival of C: Vehicle (35 days); AraC (52 days); QC (42.5 days); and AraC+QC (70 days). Median survival of D: Vehicle (32 days); AraC (55 days); QC (47 days); and AraC+QC (65 days). **E**, **F** QC fails to improve survival of secondary transplanted recipients. hCD45^+^ cells from the primary recipients of the same donor (**C**, **D**) were sorted and pooled, and transplanted into sublethally irradiated NSGS mice. Survival of recipients transplanted with ALL2 (**E**) or ALL5 (**F**) cells were monitored and plotted by the Kaplan–Meier method (ALL2: Ctr, *n* = 10; V, *n* = 12; AraC, *n* = 12; QC, *n* = 12; AraC + QC, *n* = 12; ALL5: Ctr, *n* = 10; V, *n* = 12; AraC, *n* = 13; QC, *n* = 12; AraC + QC, *n* = 12). V, Vehicle; A, AraC; QC: Quinacrine; A + QC, AraC + Quinacrine. Median survival of E: Vehicle (25 days); AraC (52 days); QC (37.5 days); and AraC+QC (57.5 days). Median survival of F: Vehicle (25 days); AraC (52 days); QC (35 days); and AraC+QC (55 days). Statistics were performed in the indicated groups: two-sided paired *t* test (parametric). Animal survival data were analyzed by Gehan–Breslow–Wilcoxon test; *p* values are indicated in Source Data files (*p < 0.05; **p < 0.01; ***p < 0.001; ****p < 0.0001).

To substantiate the in vitro findings, we next examined whether the combo AraC + QC therapy was also effective in primary ALL patient cell-xenografted mice. A cohort of primary ALL samples (Supplementary Table S2) were used for transplanting humanized NSGS mice^36,37,41,42^ followed by the treatment regimen illustrated in Fig. 4A. BM aspirates of the mice transplanted with primary human ALL cells at 8-week post transplantation showed a significant decrease of engrafted leukemic cells in AraC-treated mice in all 7 ALL patient samples tested, as compared to vehicle control or QC-treated mice (Fig. 4B, Supplementary Fig. 4A). The addition of QC to the AraC regimen further decreased leukemic burden in the recipient BM of 6 out of the 7 ALL samples compared to AraC alone (Fig. 4B, Supplementary Fig. 4A). Furthermore, the recipient mice treated with vehicle or single-agent AraC or QC died of leukemia within 80 days; whereas mice treated with the combo AraC + QC regimen showed significantly longer survival (Fig. 4C, D, Supplementary Fig. 4B). This indicates that the AraC + QC combination produces an enhanced anti-leukemic effect in primary recipients.

We then performed secondary transplantation to determine whether the combo AraC + QC regimen was effective in suppressing the long-term repopulating capacity of ALL LSCs. We retrieved donor cells from primary recipient mice transplanted with ALL2 and ALL5 cells and transplanted one million hCD45^+^ cells into each secondary recipient NSGS mouse. We observed no significant improvement in the survival of recipient mice receiving the combo AraC + QC treatment, compared to the AraC-treated group (Fig. 4E, F, Supplementary Fig. 4C). Together, these results indicate that QC enhances the anti-ALL activity of AraC in primary transplanted recipients but is less effective in inhibition of the ALL LSCs with long-term repopulating ability.

### QC promotes autophagic cell death, increases mitochondrial ROS, and inhibits NF-κB activation in AraC-resistant ALL cells

**Fig. 5:**
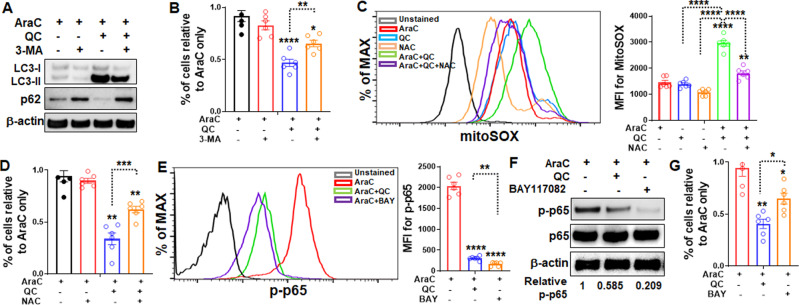
QC promotes autophagic cell death, increases mitochondrial ROS, and inhibits NF-κB in AraC-resistant ALL cells. **A** QC promotes autophagic activity in Molt4-Luc2 cells. AraC-resistant Molt4-Luc2 cells were treated with the indicated agents for 24 h followed by whole-cell lysates (WCL) extraction and western blotting analysis using antibodies against LC3A/B, p62, or β-actin. **B** Autophagy inhibitor, 3-MA partially abrogates QC-induced cell death. Cells described in (**A**) were enumerated after 24 h of treatment with the indicated agents (*n* = 6). **C** QC increases the production of mitochondrial ROS in ALL cells. AraC-resistant Molt4-Luc2 cells were treated with the indicated agents for 24 h followed by Flow cytometry analysis for mitochondrial ROS. Representative histogram (Left) and quantification (Right) are shown (*n* = 6). **D** Blocking ROS partially abolishes QC-induced cell death. Cells described in (**C**) were enumerated after 24 h of treatment with the indicated agents (*n* = 6). **E**, **F** QC inhibits NF-κB activation in AraC-resistant ALL cells. AraC-resistant Molt4-Luc2 cells were treated with the indicated agents followed by flow cytometry (**E**, *n* = 6) or western blotting analysis using antibodies against phospho-p65, p65, or β-actin (**F**). Quantification of the obtained western blots was performed by densitometry on ImageJ. Relative p-p65 are shown. **G** Cells described in (**E**) were enumerated after 24 h of treatment with the indicated agents (*n* = 6). Statistics were performed in the indicated groups: two-sided paired *t* test (parametric); *p* values are indicated in Source Data files (*p < 0.05; **p < 0.01; ***p < 0.001; ****p < 0.0001).

Several mechanisms of action for QC have been proposed in solid tumors. For example, it has been shown that QC promotes autophagic cell death and chemosensitivity in ovarian cancer and attenuates tumor growth^25^. Inhibition of NF-κB by QC is known to be cytotoxic to human colon carcinoma cell lines^43^. Another recent study shows that QC has promising anti-cancer potential against non-small cell lung cancer cells via reactive oxygen species (ROS)-mediated apoptotic signaling^44^. However, no mechanistic studies have been done for the role of QC in hematologic malignancies, including ALL cells. To elucidate the mechanisms of QC on AraC resistance in ALL, we tested these three pathways. First, we determined the QC on autophagy in AraC-resistant ALL cells. Immunoblotting analysis indicated a significantly increased autophagic activity in AraC + QC treated cells compared to those treated with AraC alone, as evidenced by the accumulation of autophagy marker, LC3-II (Fig. 5A). Consistent with the previous report that QC induces autophagic clearance of autophagy substrate, p62^25^, we observed a reduction in p62 level in AraC + QC treated cells compared to cells treated with AraC alone (Fig. 5A). Conversely, the pharmacological autophagy inhibitor, 3-Methyladenine (3-MA)^45^ prevented the LC3-II accumulation and p62 clearance in QC-treated cells (Fig. 5A), and partially abolished the sensitizing effect of QC on AraC-induced cytotoxicity in resistant ALL cells (Fig. 5B). Second, QC treatment significantly increased mitochondria ROS in AraC-resistant cells than those treated with AraC alone (Fig. 5C), as detected by a flow cytometry-based mitoSOX staining assay. Strikingly, this increased accumulation of ROS was specific in cells treated with AraC + QC (green line in Fig. 5C) and was not observed in cells treated with QC alone (blue line in Fig. 5C), suggesting that QC may act in concert with AraC to induce ROS production. Treatment with the ROS scavenger, NAC^46^, abolished the QC-induced ROS increase in resistant ALL cells (Fig. 5C), and significantly increased cell survival (Fig. 5D). Finally, QC inhibited constitutive NF-κB activation in the resistant ALL cells when combined with AraC, similar to the effect of BAY11-7082, an NF-κB inhibitor^47^, as determined by both flow cytometry (Fig. 5E) and immunoblotting (Fig. 5F). Functionally, combo AraC+QC significantly reduced ALL cell survival compared to AraC alone, mirroring the cytotoxic effect of BAY11-7082 (Fig. 5G). Together, these data suggest that the mechanisms of action for QC on overcoming AraC resistance in ALL cells are possibly through promoting autophagic cell death, increasing mitochondria ROS and inhibiting NF-κB activation.

### CASIN enhances the eradication of ALL LSCs by AraC + QC and prolongs the survival of secondary recipients

**Fig. 6:**
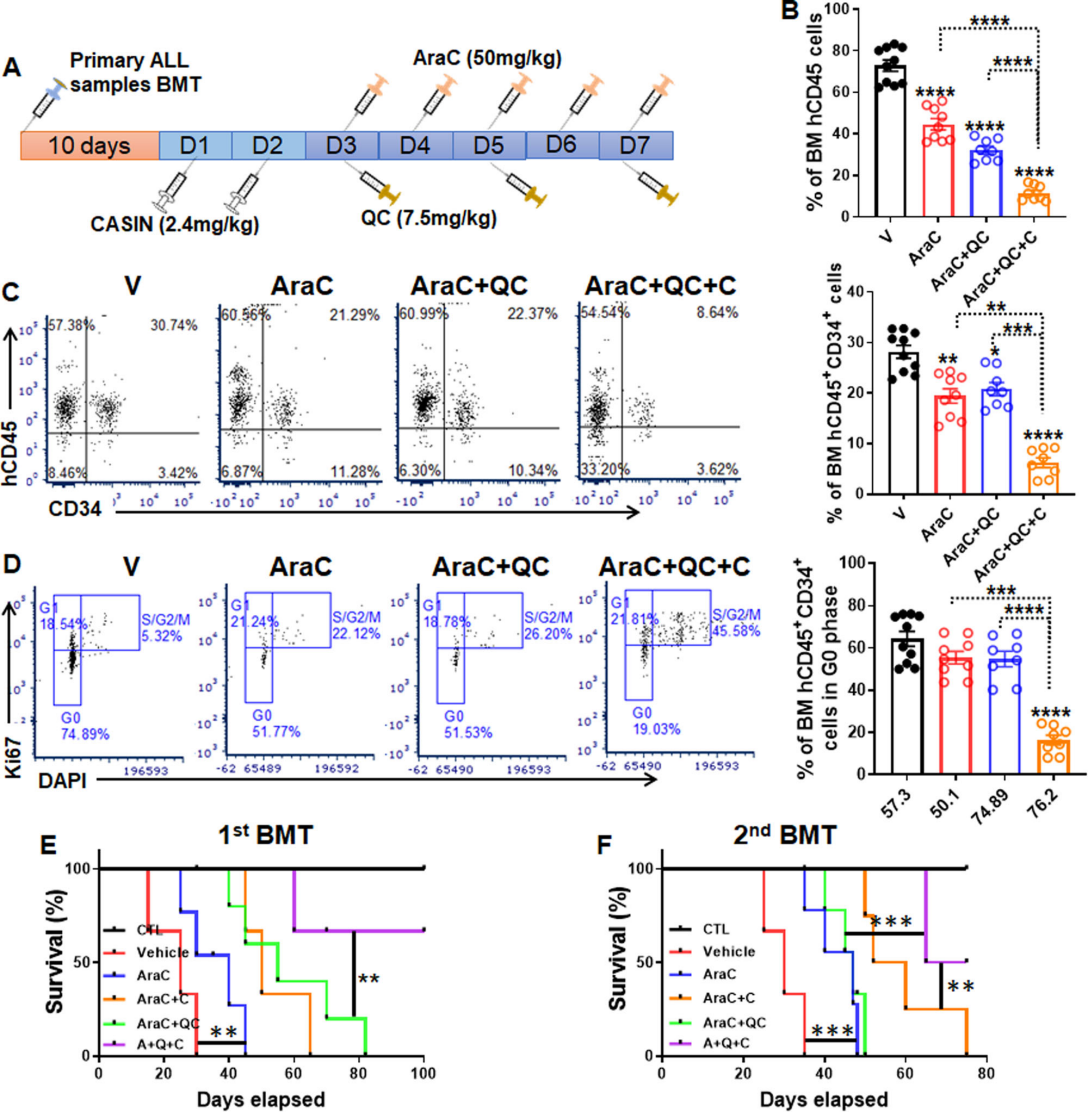
CASIN enhances the eradication of ALL LSCs by AraC + QC and prolongs the survival of recipients. **A** Schematic presentation of experimental design. **B** *C*dc42 *A*ctivity *S*pecific *In*hibitor, CASIN decreases total hCD45^+^ cells in the BM of primary recipients. 1–2 × 10^6^ primary ALL cells from pooled samples of ALL 1, 4, 7, 8 were transplanted into sublethally irradiated NSGS mice followed by the treatments described in (**A**). BM cells from the recipients were subjected to flow cytometry analysis for hCD45 (V, *n* = 10; AraC, *n* = 9; AraC + QC, *n* = 8, CASIN + QC + AraC, *n* = 8). **C** CASIN decreases LSC-enriched cell population in the BM of primary recipients. BM cells from recipients described in (**B**) were subjected to flow cytometry analysis for hCD45 and hCD34 (V, *n* = 10; AraC, *n* = 9; AraC + QC, *n* = 8, CASIN + QC + AraC, *n* = 8). **D** CASIN reduces quiescent leukemia stem cells (LSC)-enriched ALL cells in the BM of the primary recipients. BM cells from recipients described in (**C**) were gated for cell cycle analysis (V, *n* = 10; AraC, *n* = 9; AraC + QC, *n* = 8, CASIN + QC + AraC, *n* = 8). **E**, **F** CASIN improves survival of both primary and secondary recipients. BM cells from recipients of the same donor described in (**B**) were pooled and transplanted into sublethally irradiated NSGS recipients. Survival of the primary recipients (**E**; Ctr, *n* = 8; V, *n* = 9; AraC, *n* = 12; AraC + QC, *n* = 10; AraC + CASIN, *n* = 9; CASIN + QC + AraC, *n* = 11) and secondary recipients (**F**; Ctr, *n* = 10; V, *n* = 9; AraC, *n* = 8; AraC + QC, *n* = 8; AraC + CASIN, *n* = 8; CASIN + QC + AraC, *n* = 10) were monitored and plotted by the Kaplan–Meier method. Mice without transplantation served as controls (CTL). V, Vehicle; AraC, AraC; QC: Quinacrine; C, CASIN; AraC+QC, AraC + Quinacrine; AraC+QC + C, CASIN + AraC + Quinacrine. See also Supplementary Fig. 7. Statistics were performed in the indicated groups: two-sided paired *t* test (parametric). Animal survival data were analyzed by Gehan–Breslow–Wilcoxon test; *p* values are indicated in Source Data files (*p < 0.05; **p < 0.01; ***p < 0.001; ****p < 0.0001).

The observation that combo AraC + QC treatment prolonged the survival of primary transplanted recipients but exhibited limited efficacy in secondary recipients suggested that a population of niche-protected quiescent LSCs or LICs might have escaped the combo AraC + QC treatment. We thus proposed that chasing LSCs or LICs out of the protective BM niche could improve the cytotoxic effect of the combo AraC + QC regimen in ALL therapy. We took advantage of the Cdc42 inhibitor CASIN, an HSC mobilization agent that we recently identified^31,32^. CASIN inhibits intracellular Cdc42 activity specifically and transiently to induce murine and human HSPC egress from the BM to PB by suppressing actin polymerization, adhesion, and directional migration of stem/progenitor cells^31,32^. We found that CASIN did not enhance the toxicity of the AraC + QC regiment on either AraC-resistant Molt4-Luc2 cells or primary ALL samples (Supplementary Fig. 5). Since cytokine G-CSF, and the chemical antagonist of CXCR4, AMD3100 have been utilized in mobilizing HSCs for transplantation^48,49^, we compared the mobilization capacity of CASIN to the two mobilization agents in our human ALL xenotransplant model. Consistent with previous findings that CASIN-mobilized HSCs are superior in functionality to those mobilized by AMD3100^31^, we observed that while all three agents were able to mobilize total human hCD45^+^ cells from BM to PB efficiently, CASIN was superior to the other two agents in mobilizing LSC-enriched hCD45^+^CD34^+^ cells (Supplementary Fig. 6). Therefore, we chose to include CASIN in the AraC+QC regimen (Fig. 6A) and found that the addition of CASIN significantly reduced total human CD45^+^ (Fig. 6B, Supplementary Fig. 7A) and hCD45^+^CD34^+^ cells (Fig. 6C, Supplementary Fig. 7B) in the BM of the primary recipients transplanted with ALL cells, as compared to either AraC or AraC + QC group. Importantly, the inclusion of CASIN significantly reduced quiescent hCD45^+^CD34^+^ ALL cells compared to AraC or AraC + QC group (Fig. 6D, Supplementary Fig. 7C). Furthermore, CASIN greatly improved the survival of both primary (Fig. 6E, Supplementary Fig. 7D) and secondary transplanted recipients treated with AraC + QC (Fig. 6F, Supplementary Fig. 7E). Together, these data suggest that the combination of CASIN with the AraC + QC regimen eliminates quiescent LSCs and prolong the survival of xenografted mice.

### CASIN mobilizes ALL LSCs from the BM into the PB

**Fig. 7:**
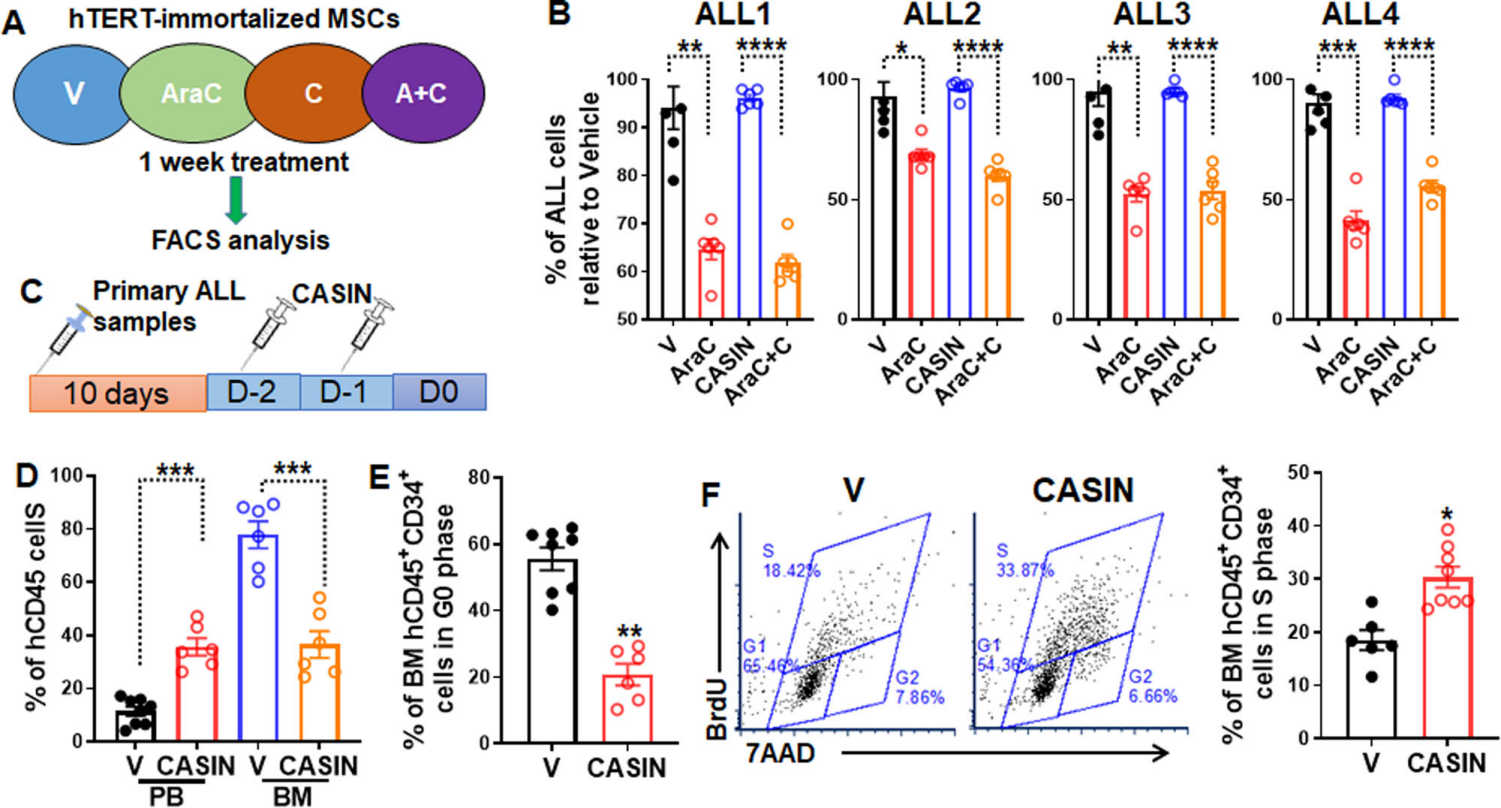
CASIN mobilizes LSC-enriched ALL cells from the BM to PB. **A** Schematic presentation of experimental design. **B** CASIN does not further increase the cytotoxic effect of AraC on primary ALL cells in vitro. Primary ALL cells were cultured on hTERT-immortalized MSCs in the presence or absence of AraC or AraC + CASIN (AraC, 2 μM; CASIN, 5 μM). Cultured cells were enumerated using counting beads and normalized to the Vehicle controls. Results are means ± SEM of three independent experiments (*n* = 6). **C** Schematic presentation of in vivo experimental design. **D** CASIN increases donor CD45^+^ cells in PB and reduces hCD45^+^ in the bone marrow (BM) of transplanted recipients. Primary ALL cells were transplanted into sublethally irradiated NSGS mice followed by two doses of CASIN injection. Cells from PB (Left) or BM (Right) were subjected to Flow cytometry analysis for hCD45 staining 1-day post treatment. Results are means ± SEM of three independent experiments (PB: V, *n* = 8; CASIN, *n* = 6; BM: V, *n* = 6; CASIN, *n* = 6). **E** CASIN decreases quiescent LSC-enriched ALL cells in the BM of transplanted recipients. BM cells described in (**D**) were subjected to Flow cytometry analysis for cell cycle 1 day post treatment. Results are means ± SEM of three independent experiments (PB: V, *n* = 8; CASIN, *n* = 6; BM: V, *n* = 6; CASIN, *n* = 6). **F** CASIN increases cycling LSC-enriched ALL cells in the BM of transplanted recipients. BM cells described in (**D**) were subjected to cell cycle analysis 1-day post treatment. Results are means ± SEM of three independent experiments (V, *n* = 6; CASIN, *n* = 8). V, Vehicle; AraC, AraC; C, CASIN; AraC + C, AraC + CASIN. Statistics were performed in the indicated groups: two-tailed, paired *t* test (parametric); *p* values are indicated in Source Data files (*p < 0.05; **p < 0.01; ***p < 0.001; ****p < 0.0001).

We previously showed that CASIN effectively and transiently mobilizes xenografted human CD34^+^ HSPCs from BM to PB in a human xenotransplant model^31^. To demonstrate that the mechanism by which CASIN promotes the eradication of ALL LSCs by AraC + QC and prolongs the survival is through its stem cell-mobilization activity, we first tested whether CASIN enhanced AraC toxicity on primary ALL cells in vitro (Fig. 7A). CASIN did not exhibit detectable cytotoxicity alone or have a more cytotoxic effect on primary ALL cells treated with AraC (Fig. 7B). We then determined the effect of CASIN on donor cell mobilization in the ALL xenotransplant model. CASIN significantly increased the percentage of hCD45^+^ ALL cells in the PB of the recipient mice compared to the vehicle control (Fig. 7C). Concomitantly, CASIN markedly decreased hCD45^+^ ALL cells in the BM of the transplanted recipients (Fig. 7D). Moreover, CASIN significantly reduced the quiescent BM LSC-enriched hCD45^+^CD34^+^ cells in the BM of transplanted recipients (Fig. 7E). This was associated with a profound increase in cycling hCD45^+^CD34^+^ cells in the BM of the recipients treated with CASIN (Fig. 7F). Thus, these results support the notion that CASIN enhances the eradication of ALL by AraC + QC through chasing ALL LSCs out of the protective BM niche.

### CASIN + QC + AraC treatment does not affect long-term normal hematopoiesis

**Fig. 8:**
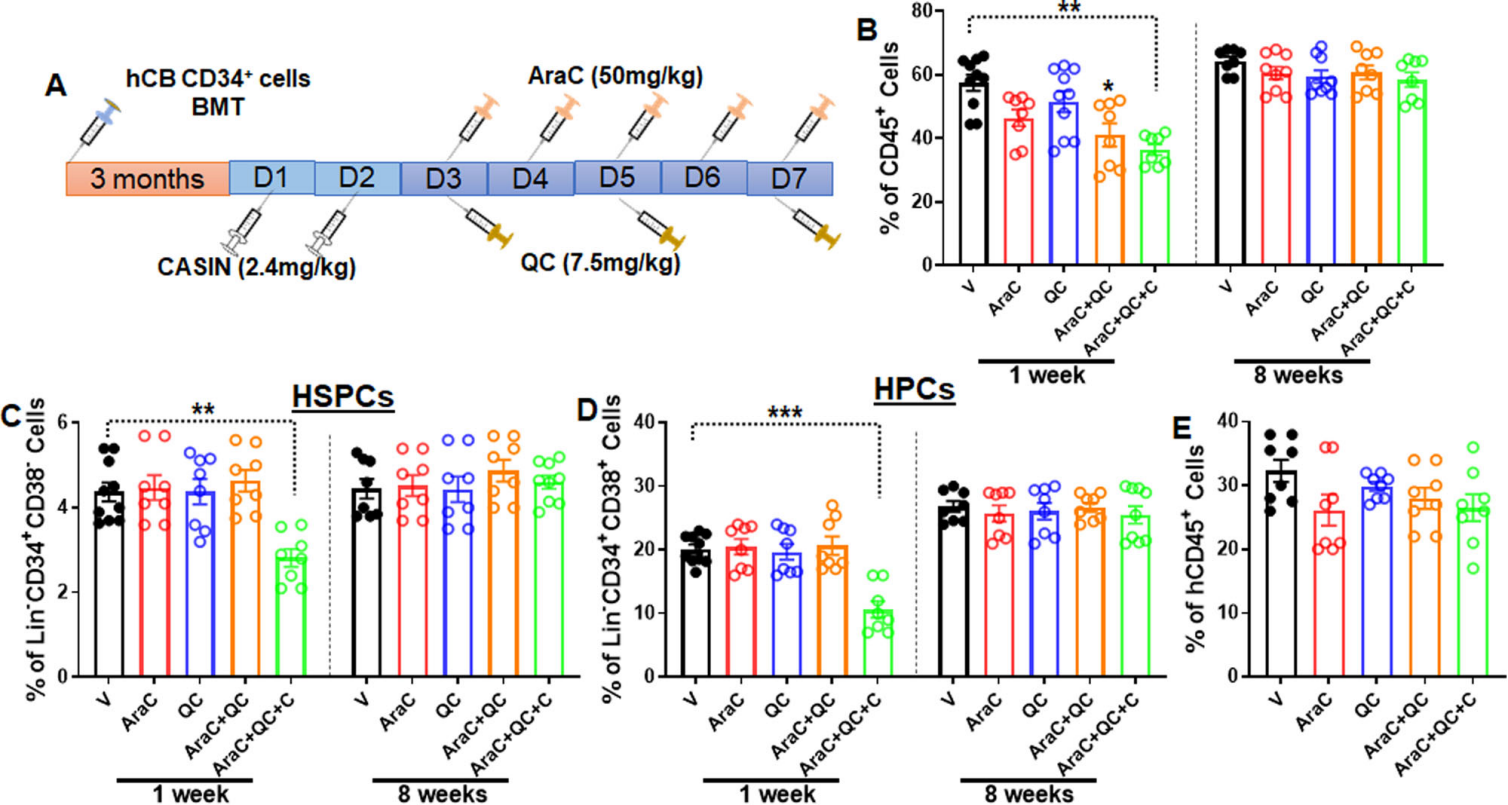
CASIN + QC + AraC treatment does not affect long-term normal hematopoiesis. **A** Schematic presentation of experimental design. **B** The effect of CASIN + QC + AraC treatment on total human engraftment in transplanted recipients. 1–3 × 10^4^ hCB CD34^+^ cells were transplanted into sublethally irradiated NSGS mice followed by the treatment described in (**A**). Percentages of human cells (hCD45^+^) in the transplanted recipients 1 week and 8 weeks post treatment were determined by flow cytometry. Results are means ± SEM of three independent experiments (Week 1: V, *n* = 10; AraC, *n* = 8; QC, *n* = 10; AraC + QC, *n* = 8; CASIN + QC + AraC, *n* = 8; Week 8: V, *n* = 8; AraC, *n* = 9; QC, *n* = 9; AraC + QC, *n* = 8; CASIN + QC + AraC, *n* = 8). **C**, **D** The effect of CASIN + QC + AraC treatment on human HSPCs. BM cells were aspirated from the transplanted recipients described in (**B**), and subjected to Flow cytometry analysis for HSPCs (hCD45^+^CD34^+^CD38^−^ cells; **C**) and HPCs (hCD45^+^CD34^+^CD38^+^ cells; **D**) 1 week and 8 weeks post treatment. Results are means ± SEM of three independent experiments (Week 1: V, *n* = 10; AraC, *n* = 8; QC, *n* = 8; AraC + QC, *n* = 8; CASIN + QC + AraC, *n* = 8; Week 8: V, *n* = 8; AraC, *n* = 8; QC, *n* = 9; AraC + QC, *n* = 8; CASIN + QC + AraC, *n* = 9). **E** CASIN + QC + AraC treatment does not affect long-term repopulating capacity of human HSPCs. BM cells from the primary recipients described in (**B**) were pooled for secondary transplantation to sublethally irradiated NSGS mice. Human engraftment was determined by Flow cytometry. Results are means ± SEM of three independent experiments (*n* = 8 per group). V, Vehicle; AraC, AraC; QC: Quinacrine; C, CASIN; AraC + QC, AraC + Quinacrine; AraC + QC + C, CASIN + AraC + Quinacrine. Statistics were performed in the indicated groups: two-tailed, paired *t* test (parametric); *p* values are indicated in Source Data files (*p < 0.05; **p < 0.01).

Finally, we wanted to determine whether the CASIN + QC + AraC regimen affected normal human hematopoiesis. To this end, we transplanted CD34^+^ cells from human cord blood (hCB) into sublethally irradiated NSGS mice. When ~70% human engraftment was established, the mice were treated with the regimens illustrated in Fig. 8A. Although we observed a decrease in the total number of human CD45^+^ cells 1-week post-treatment in the transplanted recipient mice treated with AraC, AraC + QC, or CASIN + QC + AraC, no significant difference in total human engraftment was found at 8 weeks post-treatment for all treatment groups compared to the vehicle controls (Fig. 8B). Furthermore, we found that while CASIN + QC + AraC treatment caused a decrease in human HSPCs (Lin^−^hCD34^+^CD38^−^) and more mature HPCs (Lin^−^hCD34^+^CD38^+^) at 1-week post treatment, probably due to the transient mobilization of these cells by CASIN from the BM to PB, comparable numbers of HSPCs and HPCs were observed at 8 weeks post-treatment in the transplanted recipients of all groups (Fig. 8C, D). More importantly, the serial transplantation assay showed that total human engraftment (hCD45^+^) in secondary recipients was not affected by the treatment of AraC, QC, AraC + QC, or CASIN + QC + AraC (Fig. 8E). Thus, these results suggest that the CASIN + QC + AraC regimen does not affect long-term normal hematopoiesis.

## Discussion

Despite significant progress in the introduction of molecularly-targeted therapies and innovative immunotherapeutic approaches, the outcome in relapsed pediatric ALL therapy is not satisfactory^5^. By employing ex vivo small molecule screening, here we identified QC as a drug that can sensitize the standard AraC-mediated chemotherapy in ALL. When combined with the stem cell mobilizer, CASIN, QC possesses a significant chemosensitizing activity that overcomes AraC resistance in human ALL by eradicating the niche-protected quiescent LSCs and preventing relapse in a human xenotransplant model. There are several findings that highlight the significance of our studies: (1) QC enhances the cytotoxicity of AraC in resistant ALL cell line in vitro; (2) QC delays ALL development but fails to prevent ALL relapse in vivo; (3) QC enhances AraC response on primary ALL cells in vitro, but does not have anti-ALL effect on L-LTC-ICs; (4) QC + AraC treatment prolongs the survival of primary transplanted recipients but not in secondary recipients; (5) QC promotes autophagic cell death, increases mitochondria ROS and inhibits constitutive NF-κB activation of AraC-resistant ALL cells; (6) CASIN facilitates the eradication of ALL LSCs by AraC + QC and prolongs the survival of both primary and secondary recipients without affecting long-term normal human hematopoiesis; (7) the mechanism of CASIN in facilitating the eradication of ALL LSCs by AraC + QC is through its mobilizing capacity of the LSCs from the BM to PB.

Although QC was identified in an AraC-resistant ALL cell screen, we found that QC could also enhance the cytotoxic effect of AraC on ALL cells in both culture and xenotransplanted mice. This chemo-potentiation function is not only beneficial for increased clearance of ALL but also has implications for an additive effect on treatment paradigms for elderly patients who are intolerant to high chemotherapy doses. In support of this notion, our effective 5-day treatment regimen of AraC (50 mg/kg) combined with intermittent dosing of the QC (7.5 mg/kg) in our xenograft models not only mimics the AraC exposures reported in the clinic^50^ but also is significantly lower than previously published in vivo QC dosages^51–54^. Although several in vitro studies suggest a potential of QC in treating AML^23,26,55–58^, whether QC has an effect in other leukemia, especially in refractory ALL remains unknown. Thus, our present study in ALL lends support for the utility of QC as a chemo-potentiation strategy for leukemia therapy. Given that AraC is a commonly used anti-leukemia drug for the treatment of other leukemias, including AML, it may be of interest to explore the potential of QC in AML treatment.

Another interesting finding of the current study is that while QC could overcome AraC resistance in a short-term manner in our ALL xenograft models of both cell lines (Fig. 2) and primary patient samples (Fig. 4), combo AraC+QC failed to prolong the survival of secondary transplanted recipients (Figs. 4 and 5). This suggests that QC could potentiate the clearance of the bulk ALL cells by AraC, but has less effect on AraC-mediated LSC eradication. Indeed, both limited dilution and patient-derived xenograft assays showed that AraC + QC failed to reduce LSC-enriched ALL L-LTC-ICs and hCD45^+^CD34^+^ cells (Figs. 3 and 5). These data are consistent with many previous studies showing that LSCs can escape intensive chemotherapy^59–62^. It is now accepted that LSCs are the source of chemoresistance and relapse and that elimination of LSCs is crucial for long-term cure in leukemia therapy. In this context, our approach to improving the efficacy of the combo AraC + QC treatment utilized a Cdc42 inhibitor (CASIN), which effectively induced LSCs mobilization and exposed them to the killing by AraC+QC. We recently showed that targeting Cdc42 by CASIN presents a utility for stem cell mobilization^31,32^. We propose that this stem cell mobilizer may prove valuable in overcoming the chemoresistance of relapse-inducing LSCs and improving survival in relevant models of human acute leukemia.

A major challenge in chemotherapy is optimal dosing/regimen that is both effective and less harmful to normal cells. Although our CASIN + QC + AraC regimen caused a reduction of normal human HSPCs and HPCs in the BM of the transplanted recipients after 1 week, we observed no decrease in the number of HSPCs and HPCs at 8-week post treatment (Fig. 7). We attribute this short-term effect to the mobilization of these precursor cells from the BM to the PB by CASIN, which can transiently and reversibly induce HSPC egress from the BM^31,32^. In addition, although CASIN + QC + AraC treatment reduced short-term human engraftment, we observed no difference in human engraftment in secondary transplanted recipients compared to the vehicle controls. We thus conclude that the CASIN + QC + AraC regimen has minimal effect on long-term normal hematopoiesis. These data also suggest that the CASIN + QC + AraC regimen acts primarily on the more actively cycling cells, as opposed to the quiescent components of the HSPC compartment. Consistent with this notion, our results in ALL cytotoxicity assay and LDA (Fig. 3) show that AraC + QC inhibited the primary ALL blasts in vitro, but does not have an anti-ALL effect on L-LTC-ICs.

Anti-cancer benefits of chemotherapies are considered to be a consequence of direct cytotoxicity or permanent arrest of the cell cycle machinery^63^. Many mechanisms of action have been proposed in different drugs in treating a variety of cancers. For example, autophagy is known to function as a death execution to induce autophagic cell death in response to chemotherapy^64^ and has been proposed as a mediator of chemotherapy-induced cell death in cancer^64^. NF-κB is a key transcription factor, playing crucial roles in the development and progression of cancer and chemoresistance through the activation of a multitude of mediators including anti-apoptotic genes^65^. Since activation of NF-κB has been linked to various cellular processes in cancer, including inflammation, transformation, proliferation, angiogenesis, invasion, metastasis, chemoresistance, and radiodensities^66^, it has emerged as a promising anti-cancer target^65^. Most chemotherapeutics also elevate intracellular ROS levels, alter redox-homeostasis of cancer cells, therefore inducing ROS-mediated cell injury in cancer^67^. Here, we show in hematologic malignancies that QC enhances AraC-mediated cytotoxicity in ALL through multiple mechanisms, which highlights the potential utility of QC in combination with other chemotherapeutic agents currently used in leukemia therapy. Specifically, we show that QC promoted autophagic cell death, increased mitochondria ROS production, and inhibited NF-κB activation in AraC-resistant ALL cells. These findings suggest that QC may act in concert with AraC to induce ALL cell death and highlight the therapeutic potential of QC in chemoresistant ALL, as well as other leukemias.

In summary, we identified QC as a chemosensitizing agent, and demonstrate that the QC-CASIN combination is a promising strategy to eradicate LSCs and overcome chemoresistance in ALL treatment.

## Methods

### Mice, cell lines, and reagents

Humanized NSGS mice expressing transgenic cDNA encoding human SCF, GM-SCF, and IL-3 (NOD SCID *IL-2Rγ*^−*/*−^ SCF, GM-CSF, and IL-3)^36^, were purchased from Jackson Laboratories (Stock # 013062; Jackson Laboratory, Bar Harbor, ME). Both male and female mice at 8–10 weeks of age were used. All animal experiments were carried out in accordance with the National Institutes of Health Guidelines for the Care and Use of Laboratory Animals and approved by the Institutional Animal Care and Use Committee of the University of Pittsburgh (IACUC protocol # 21069196).

Human ALL cell line Molt4-Luc2 expressing luciferase maker was purchased from America Type Culture Collection (ATCC; Manassas, VA) and cultured in RPMI1640 medium supplemented with 10% FBS.

AraC (Cytosine β-D-arabinofuranoside; C1768, Sigma-Aldrich, St Louis, MO) was used at 50 mg/kg concentration for in vivo^37^ and at 2 μM concentration for in vitro experiments. Quinacrine dihydrochlride (QC) was purchased from Sigma-Aldrich (Sigma-Aldrich, St Louis, MO). For in vitro experiments, aliquots of QC (100 μM) were stored at −20 °C and used at a final concentration of 100 nM. For in vivo experiments, aliquots of QC (10 mg/ml) were stored at −20 °C and diluted in 100 mM sodium citrate buffer immediately prior to each experiment^51–53^. QC was used at 2 mg/ml via Intraperitoneal (i.p.) injection to reach the final concentration of 7.5 mg/kg. QC was administered to the experimental mice every 2 days for 3 times.

Autophagy inhibitor, 3-Methyladenine (3-MA) were obtained from Fisher Scientific (Hampton, NH) and used at 10 mmol/L^45^. ROS scavenger, NAC (N-acetyl-l-cysteine; Sigma-Aldrich, St Louis, MO) was used at 10 nM^46^. BAY11-7082 (Fisher Scientific, Hampton, NH) was used at 10 μM for 6 h^68^. CASIN (*C*dc42 *A*ctivity *S*pecific *In*hibitor) were purchased from Sigma-Aldrich (St Louis, MO). For in vitro studies, CASIN (containing 0.02-0.2% DMSO) was used at 5 μM in the culture medium. For in vivo experiments, CASIN was dissolved in PBS with 15% ethanol and administered by i.p. (2.4 mg/kg) injection. Separately, mice were treated with G-CSF (100 μg/kg per day once a day for 5 days, IP) or AMD3100 at 5 mg/kg by i.p. injection^31^.

### Establishment of AraC resistant cells

To develop AraC resistant cells, Molt4-Luc2 cells were treated with increasing concentrations of AraC (1 nM–2 μM)^69–71^. The cultures were observed daily and were passaged using gradually increasing concentrations of AraC. When the doubling time in the presence of 2 μM AraC was almost the same as that of the parent cell lines in the absence of AraC, the cells were saved for future use.

### LOPAC^1280^ Library

LOPAC^1280^ Library is a collection of high-quality, innovative pharmacologically active compounds that span a broad range of cell signaling and neuroscience areas, and are most commonly used to candidate new drug discover assays^33–35^. The composition of the LOPAC^1280^ library reflects the most commonly screened targets in the drug discovery community. It contains 1280 pharmacologically active Sigma-RBI compounds arrayed in 96-well format.

### Cell viability assay

Molt4-Luc2 cells cultured with AraC in the presence or absence of QC were subjected to cell viability analysis measured using the CellTiter-Glo Luminescent Cell Viability Assay Kit (Promega, Madison, WI) per the manufacturer’s instructions and a Synergy H1 hybrid multi-mode Reader (BioTek, Winooski, VT).

### In vitro culture of primary ALL cells

Primary human ALL cells were obtained after informed consent at West Virginia University Cancer Center; Cincinnati Children’s Hospital Medical Center Respiration Core; and Pittsburgh Biospecimen Core under the approved Institutional Review Broad (IRB) protocols: #1310105737; STUDY19030357 and # 2011-3023. Primary human ALL cells were cultured on hTERT-immortalized primary BM mesenchymal stromal cells (MSCs)^39,40^. MSCs were seeded at a density of 10^4^ cells/cm^2^ in MSC medium 48 h prior to adding to ALL. ALL cells were seeded onto MSC at a density of 2 × 10^6^ cells/ml in SFEM II medium (StemCell Technologies, Vancouver, BC, Canada) supplemented with 20% fetal calf serum (GIBCO, Life Technologies), 20 ng/ml recombinant IL-3 (R&D Systems, Abingdon, UK) and 10 ng/ml recombinant IL-7 (R&D Systems, Minneapolis, MN). ALL cells were harvested every 7 days. Non-adherent cells present in supernatant medium were washed with PBS and passed through a 15 μm filter (pluriSelect Life Science, Leipzig, Germany). ALL cells were separated from MSCs by magnetic cell separation using hCD45 microbeads (Miltenyi Biotec, Auburn CA). Viable ALL cells were counted by Trypan blue exclusion, re-suspended in fresh ALL medium, and seeded onto fresh MSC.

### Establishment of ALL models in immunodeficient mice

Primary human ALL cells and human cord blood (hCB) CD34^+^ cells were obtained after informed consent at West Virginia University Cancer Center; Cincinnati Children’s Hospital Medical Center Respiration Core; and Pittsburgh Biospecimen Core under the approved Institutional Review Broad (IRB) protocols: #1310105737; STUDY19030357 and # 2011-3023. ALL cell line (1 × 10^6^ cells/mouse), primary ALL samples (1–2 × 10^6^ cells/mouse), or human cord blood (hCB) CD34^+^ (1–3 × 10^5^ cells/mouse), were transplanted into 8–12 weeks old humanized NSGS mice (The Jackson Laboratory, Bar Harbor, ME) using intravenous (i.v.) injection^36,37,41,42^. Twenty-four hours before transplantation, mice were pre-conditioned by sublethal irradiation at the dose of 2.5 Gy. To assess the level of engraftment, BM samples were aspirated from a long bone while mice were under isoflurane anesthesia, at different time points after transplantation. After the establishment of engraftment, mice were randomized into different treatment groups and treated with drugs according to the experimental design (Vehicle, PBS with 15% ethanol; AraC, 50 mg/Kg; QC, 10 mg/kg; AraC + QC, a combination of QC with AraC).

For secondary transplant, mouse BMs of the same experimental condition were pooled and human CD45^+^ cells were sorted. Totally, 1 × 10^6^ cells were transplanted into secondary sublethally irradiated NSGS mice. Ten days post-BMT, the recipient mice were randomly divided into 4 groups (*n* = 10–12 mice/group) and treated with the indicated drugs (Vehicle, PBS with 15% ethanol; AraC, 50 mg/kg; QC, 10 mg/kg; AraC + QC, a combination of QC with AraC). The survival outcome of the mice was monitored and recorded.

To determine whether CASIN + QC + AraC treatment affects normal hematopoiesis, 1–3 × 10^4^ hCB CD34^+^ cells were transplanted into sublethally irradiated NSGS mice followed by the indicated treatment. All human-NSGS xenograft studies were carried out in accordance with the National Institutes of Health Guidelines for the Care and Use of Laboratory Animals and approved by the Institutional Animal Care and Use Committee of the University of Pittsburgh (IACUC protocol # 21069196).

### Bioluminescence imaging

Isofluorane-anesthetized animals were imaged using the Xenogen IVIS imaging system 5–10 min after d-luciferin (Caliper Life Sciences, Waltham, MA) was injected intraperitoneally (i.p., 150 mg/kg). Five minutes after injection of the d-Luciferin, images were acquired for 30 s to 2 min using Living Image analysis and acquisition software 4.7 (Caliper Life Sciences, Waltham, MA) from both ventral and dorsal sides of the mice. The photons emitted from Molt4-Luc2 expressed as Flux (photons/s/cm^2^/steradian), were quantified and analyzed using the “Living image” Pro. 2.0 software (Caliper Life Science, Waltham, MA)^38^.

### Flow cytometry analysis

For analysis and sorting of ALL and HSPCs derived from human Cord Blood or adult BM, cells were stained with hCD45-PE Cy7 (Clone: H30, Cat: 560915; BD Biosciences, San Jose, CA), mCD45-PerCP Cy5.5 (Clone:30-F11, Cat: 550994; BD Biosciences, San Jose, CA), Lineage-FITC (Lin1, Cat: 340546; BD Biosciences, San Jose, CA), CD34-PE (Clone 581, Cat: 560941, dilution: 1 in 25; BD Biosciences, San Jose, CA), and CD38-APC (Clone HIT2, Cat: 555462; BD Biosciences, San Jose, CA). Human grafts in mice were assessed using CD19-FITC (Clone: H1B19; Cat: 555412; BD Biosciences, San Jose, CA), CD3-APC (Clone: UCHT1, Cat: 561811; BD Biosciences, San Jose, CA), hCD45-PeCy7 (Clone: H30, Cat: 560915; BD Biosciences, San Jose, CA), and mCD45PerCPCy5.5 (Clone:30-F11, Cat: 550994; Biosciences, San Jose, CA). Non-viable cells were excluded by DAPI staining. Appropriate isotype-matched antibodies were used as controls. Flow cytometry analysis was performed using an LSRII flow cytometer (BD Biosciences, San Jose, CA). Cell sorting was performed using a FACS Aria or INFLUX (BD Biosciences, San Jose, CA). FACSDiva software v 6.1.3 was used for data acquisition (BD Biosciences, San Jose, CA). Flow cytometry data were analyzed using FCSExpress software 7.08.0018 (De Novo Software, Pasadena, CA).

Cells were subjected to the indicated mice followed by flow cytometry analysis to determine cell apoptotic status following Annexin V and 7-AAD staining, or cell cycling analysis using Ki67 and DAPI staining.

For BrdU incorporation assay, Bromodeoxyuridine (BrdU, 150 μl of 10 mg/ml) were intraperitoneally (i.p.) injected to subjected mice followed by BM cells isolation 14 h later. BrdU incorporated cells (S phase) were analyzed with the APC BrdU Flow Kit (BD Biosciences, San Jose, CA), following the manufacturer’s instructions. Briefly, cells were surface stained then fixed and permeabilized using BD Cytofix/Cytoperm Buffer. After 1 h incubation with DNase at 37 °C, cells were stained with APC-conjugated anti-BrdU monoclonal antibody. 7-aminoactinomycin (7-AAD) was added to each sample right before Flow Cytometry analysis (BD Biosciences, San Jose, CA).

Mitochondrial ROS staining procedure was adapted from the previous report^72^. Briefly, treated cells were stained with MitoSOX red (5 μM, Molecular Probes, Waltham, MA) at 37 °C for 10 min in the dark, then washed with pre-warmed PBS. Cell pellets were suspended in pre-warmed PBS followed by Flow cytometry analysis.

To detect NF-κB activation, cells were fixed and permeabilized using BD Cytofix/Cytoperm buffer followed by incubation with phospho-p65 antibody (#3033S, Clone 93H1, Cell Signaling Technology, Beverly, MA) for 30 min. Cells were then washed and incubated with Alexa Fluor 488-conjugated secondary antibody (Invitrogen, Waltham, MA) for Flow cytometry analysis.

### LTC/limiting dilution assay (LDA)

For LTC/LDA, human cells from short-term culture were magnetically sorted using hCD45 beads using the StemSep system (Stem Cell Technologies, Vancouver, BC, Canada) according to manufacturer’s instructions and plated on a freshly prepared irradiated hTERT-immortalized primary BM MSCs layer at the indicated concentrations. They were cultured in normoxic conditions with no addition of drug for 4 weeks and a half medium changes were performed once a week without disrupting the established feeder layer^40,73^. At the end of the assay, the colonies were enumerated. The L-LTC-IC frequency were calculated using an online web tool (http://bioinf.wehi.edu.au/software/elda/index.html).

### Western blot assay

Cells were washed with ice-cold PBS and lysed in an ice-cold lysis buffer containing 50 mM Tris-HCl (pH 7.4), 0.1% NP40, and 1 M NaCl supplemented with protease and phosphatase inhibitors (10 μg/ml of aprotinin, 25 μg/ml of leupeptin, 10 μg/ml of pepstatin A, 2 mM phenylmethylsulfonyl fluoride, 0.1 M NaP_2_O_4_, 25 mM NaF, and 2 mM sodium orthovanadate) for 30 min on ice. Cell debris was removed from the lysate by centrifugation. Protein lysate was resolved on sodium dodecyl sulfate-polyacrylamide gel electrophoresis and transferred onto nitrocellulose membranes. Immunoblots were then probed with primary antibodies for LC3A/B (#4108S; Cell Signaling Technology, Beverly, MA), p62 (#5114S; Cell Signaling Technology, Beverly, MA), p65 (#8242S; Cell Signaling Technology, Beverly, MA), phospho-p65 (#3033 S, Clone 93H; Signaling Technology, Beverly, MA) and β-actin (Clone AC-74, Cat # A2228, Sigma-Aldrich, St Louis, MO) for 12–16 h at 4 °C. Signals were visualized by incubation with anti-mouse secondary antibody, followed by enhanced chemiluminescence (Amersham Biosciences, Pittsburgh, PA). Western blot images were taken using Amersham Imager 680 chemiluminescence imaging system (Amersham Biosciences, Pittsburgh, PA) for both colorimetric markers and chemiluminescence images. Quantification of the obtained western blots was performed by densitometry on ImageJ 1.8.0 (Bethesda, MD).

### Statistical analysis

Graphpad Prism 9.0 (San Diego, CA) was used for all statistical analyses. Paired or unpaired student’s *t-test* was used for two-group comparisons. Survival data were plotted by the Kaplan–Meier curve method and analyzed by the Gehan–Breslow–Wilcoxon test. Results are presented as mean ± SEM. * indicates *p* < 0.05; ** indicates *p* < 0.01, *** indicates *p* < 0.001; **** *p* < 0.0001.

For other experimental procedures, see Supplementary Information.

### Reporting summary

Further information on research design is available in the Nature Research Reporting Summary linked to this article.

## Supplementary information

Supplementary Information

Reporting summary

## Data Availability

All data presented in the paper are available in the Source Data file. Source data are provided with this paper.

## Source data

Source Data
