# Supplementary material for: Quinacrine-CASIN combination overcomes chemoresistance in human acute lymphoid leukemia
Source: Nat Commun. 2021 Nov 26;12:6936. doi: 10.1038/s41467-021-27300-w (PMC8626516; doi:10.1038/s41467-021-27300-w)
Supplement: Supplementary file 1 — Supplementary Information [file 41467_2021_27300_MOESM1_ESM.pdf]

Supplementary Information

Wu *et al.*

Quinacrine-CASIN combination overcomes chemoresistance in human acute lymphoid leukemia

Supplementary table

Supplementary Table 1. Hits from the initial and second screen

Number	Compound Name	Normalized Luciferase Intensity	
		1st Screen	2nd Screen
1	Thapsigargin	0.3027	1.578358947
2	XCT790	0.467601	1.502778359
3	Trimethoprim	0.237928	1.246346142
4	Tenidap	0.0654	0.762091671
5	Ketoconazole	0.474786	1.227097019
6	AMN082	0.39587	1.154084518
7	SKF-525A Hydrochloride	0.327579	1.104988757
8	Piceatannol	0.298385	0.170133775
9	NNC 55-0396/NO287	0.410349	0.801662727
10	Nifedipine	0.372546	1.271811928
11	NEMADIPINE-A	0.094386	0.670183383
12	Olomoucine	0.323543	1.263997573
13	LY-367,265	0.048159	0.083167067
14	5-Hydroxyindol acetic acid	0.374438	1.344394282
15	Imazoda	0.223852	1.49240872
16	(+)-Hydrastine	0.271645	1.478971716
17	Serotonin hydrochloride	0.278046	1.638080388
18	Ciproxifan hydrochloride	0.353916	1.20951472
19	Primidone	0.481995	1.003576245
20	S8567/SU4312	0.360696	0.336634335

21	Terbutaline hemisulfate	0.201784	1.271112938
22	BIX 01294 trihydrochloride hydrate	0.270174	0.347505814
23	PAPP	0.323836	0.560260086
24	N-p-Tosyl-L-phenylalanine chloromethyl ketone	0.34593	0.586995832
25	TTNPB	0.22132	0.389657757
26	(±)-Taxifolin	0.307208	1.097901556
27	Tolazamide	0.493049	1.019565973
28	6-Methyl-2-pyridine hydrochloride	0.450774	1.742911039
29	Quinacrine	0.066726	0.127293082
30	Moxonidine hydrochloride	0.298385	1.016398752
31	BF-170 hydrochloride	0.327579	0.312466675
32	Morin	0.271645	1.190119654
33	Pergolide methanesulfonate	0.271645	0.35861797
34	Minoxidil	0.443541	1.011974373
35	Rufinamide	0.392237	0.89144588
36	IC261	0.34593	0.408602192
37	Putrescine dihydrochloride	0.49629	1.109359368
38	5 alpha-pregnan-3alpha-01-2-one	0.378798	0.786505131
39	Mibefradil dihydrochloride	0.053178	0.385714734
40	Lansoprazole	0.492843	1.078941759
41	Mitoxantrone	0.023849	0.548192898
42	(+)-Quisqualic acid	0.145591	0.95249361
43	BIO	0.057039	0.697121401
44	Spironolactone	0.442517	0.896039246

45	BIA 2-093	0.428903	1.21742125
Relative luciferase intensity of each hit was normalized to the untreated controls.<br>45 hits from initial screening were subjected to 2nd screening. Each compound was tested in triplicate.			

Supplementary Table 2. Summary of ALL samples used in the study

#	Subtype	Genetic aberrations
**ALL1**	T-ALL	t(4;11)(q21;q23)/MLL-AF4
**ALL2**	T-ALL	t(5;14)(q35;q32)/HOX11L2
**ALL3**	T-ALL	t(7;10)(q34;q24)/TLX1(HOX11)
**ALL4**	B-ALL	t(9;22), Ph+, BCR/ABL
**ALL5**	B-ALL	del(9)(p11.2),del(17)(p12)
**ALL6**	B-ALL	t(9;22)-BCR/ABL; triosmy 4
**ALL7**	T-ALL	add(7)(q34),t(9;22)(q34;q11.2)-BCR/ABL
**ALL8**	B-ALL	t(1;19)(q23;p13.3)/E2A-PBX1
**ALL9**	T-ALL	t(11;19)(q23;p13.3)/MLL-MLLT1(ENL)
**ALL10**	B-ALL	t(9;22), Ph+, BCR/ABL
**ALL11**	B-ALL	t(4;11)(q21;q23); partial monosomy 17p

Supplementary figures

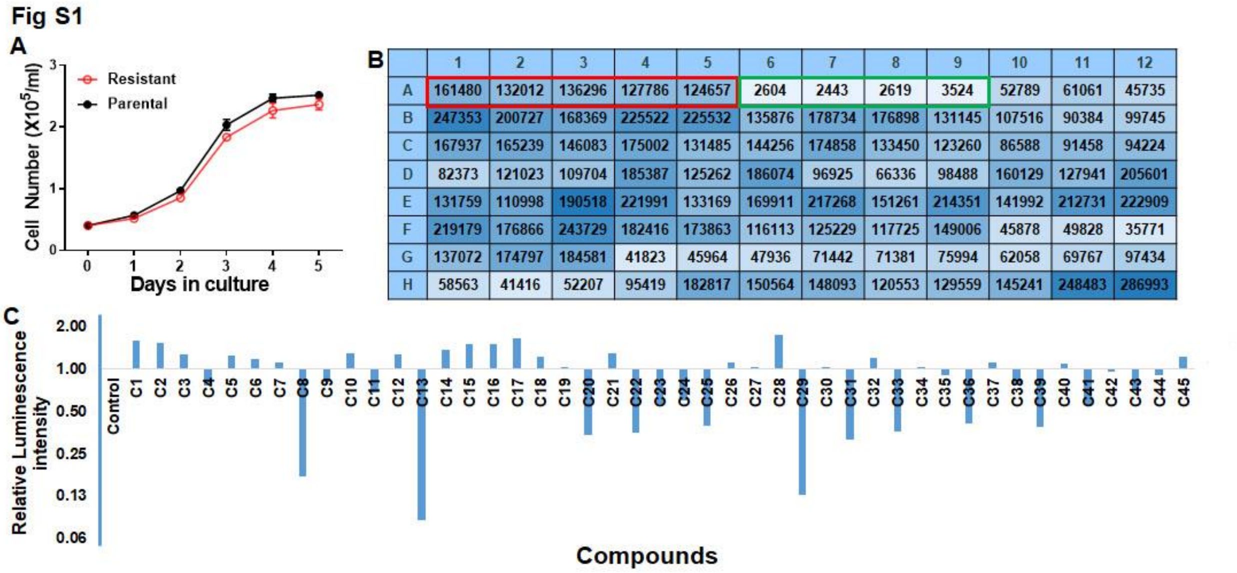

	1	2	3	4	5	6	7	8	9	10	11	12
A	161480	132012	136296	127786	124657	2604	2443	2619	3524	52789	61061	45735
B	247353	200727	168369	225522	225532	135876	178734	176898	131145	107516	90384	99745
C	167937	165239	146083	175002	131485	144256	174858	133450	123260	86588	91458	94224
D	82373	121023	109704	185387	125262	186074	96925	66336	98488	160129	127941	205601
E	131759	110998	190518	221991	133169	169911	217268	151261	214351	141992	212731	222909
F	219179	176866	243729	182416	173863	116113	125229	117725	149006	45878	49828	35771
G	137072	174797	184581	41823	45964	47936	71442	71381	75994	62058	69767	97434
H	58563	41416	52207	95419	182817	150564	148093	120553	129559	145241	248483	286993

Supplementary Figure 1. *In vitro* screening of the LOPAC^{1280} library identifies potential chemosensitizing agents. (A) Generation of AraC-resistant Molt4-Luc2 cells. Parental Molt4-Luc2 cells were cultured in the presence of increased doses of AraC (1 nM to 2 μM) for 2 months. The cultures were observed daily and were passaged using gradually increasing concentration of AraC. When the doubling time in the presence of 2 μM AraC reached the same level as that of the parent cell lines in the absence of AraC, the cells were aliquoted for future use. The data show the growth curse of an established AraC-resistant Molt4-Luc2 cell line compared to that of the parent cell line (n= 3 of technical replicates). (B) *In vitro* screen identifies potential hits. 50,000 AraC-resistant Molt4-Luc2 cells were seeded in triplicates in each well of the 96-well-plates. Each compound from LOPAC^{1280} library was added into an individual well and cultured for 24h according to manufacturer's instructions. Luciferase intensity of each well was measured by using a Synergy H1 luminescent plate reader. Potential compounds with inhibitory

effect on AraC-resistant cells were selected for future experiments. Representative luminescent data are shown. The density of blue color represents the intensity of luciferase. A1-A5: cells only; A6-A9: empty wells; A10-H12: different compounds from LOPAC[1280] Library. Each compound was tested in triplicate. (C) Further validation indicates that 18 out of 45 exhibited significant inhibitory effect on AraC-resistant Molt4-Luc2 cells. 1X10^5 AraC-resistant Molt4-Luc2 cells were seeded in triplicates in each well of the 24-well plates. Each hit (45 total) compound from initial screen was added into an individual well and cultured for 24 h. Luciferase intensity of each well were measured by using a Synergy H1 luminescent plate reader. Paired *t*-test was used for the statistical analysis. Statistics were performed in the indicated groups: Two-tailed, paired *t* test (parametric); p values are indicated in Source Data files.

Fig S2

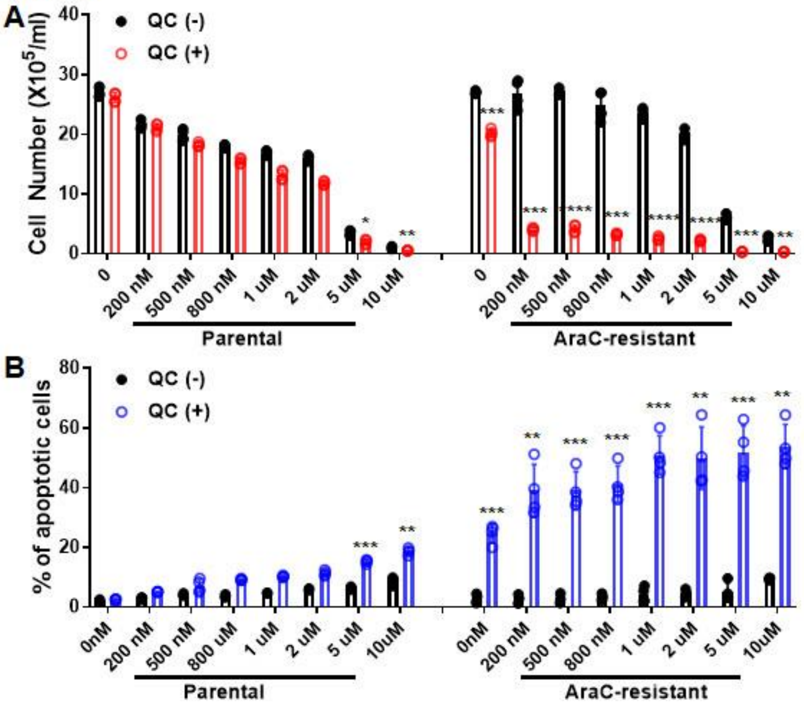

Supplementary Figure 2. QC inhibits the growth of AraC-resistant Molt4-Luc2 cells. (A) AraC-sensitive (parental) and AraC-resistant Molt4-Luc2 cells were seeded in 6-well plates for culture with different concentrations of AraC (200 nM to 10 μM) in the presence or absence of QC (100 nM). Cell numbers were enumerated and plotted at the indicated time points (n= 4). (B) Cells described in (A) were subjected to flow cytometry analysis for Annexin V and 7AAD. Statistical significance was assessed using student's *t-test* (* p<0.05; ** p<0.01; n= 4). Statistics were performed in the indicated groups: two-sided paired *t*-test (parametric); p values are indicated in Source Data files (* p<0.05; ** p<0.01; *** p<0.001; **** p<0.0001).

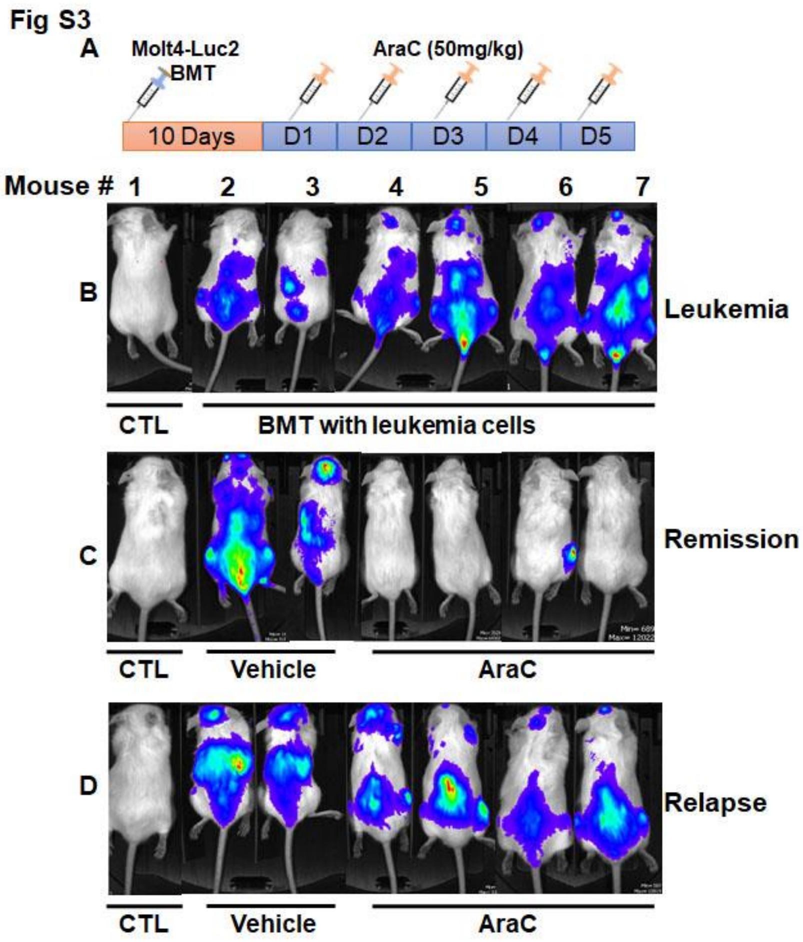

Supplementary Figure 3. Establishment of ALL remission-relapse NSGS xenograft model using Molt4-Luc2 cells. (A) Schematic presentation of experimental design. One million Molt4-Luc2 cells were transplanted into each sublethally irradiated NSGS recipient. 10 days post bone marrow transplantation (BMT), vehicle, AraC, QC or AraC+QC were administered daily to the recipients for consecutive 5 days followed by IVIS imaging analysis at the indicated time points. Mice without transplanting the Molt4-Luc2 ALL cells were served as control (CTL). The mice were randomly divided into 3 groups: (B) the Molt4-Luc2 ALL cell-transplanted mice were left without treatment and luciferase signals were imaged using the IVIS imaging system 10 days post BMT; (C) the Molt4-Luc2 ALL cell-transplanted mice were subjected to consecutive 5 days of AraC or vehicle injection, and luciferase signals were imaged 5 days post-injection; (D) the Molt4-Luc2 ALL cell-transplanted mice were subjected to consecutive 5 days of AraC or vehicle injection, and luciferase signals were imaged 15 days post-injection (n= 6/group).

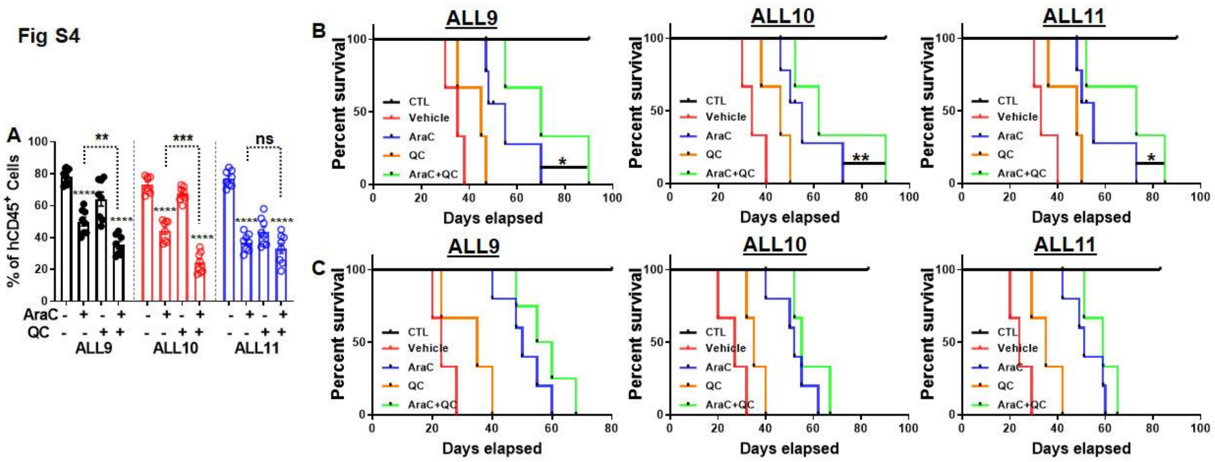

Supplementary Figure 4. QC enhances the cytotoxicity of AraC in primary ALL cells but fails to delay ALL relapse in secondary recipients. (A) QC reduces leukemia burden in primary recipients. Percentage of human cells in the bone marrow of mice

transplanted with 3 ALL patient samples were determined by flow cytometry 1 month post-transplant (ALL9: V, n= 10; AraC, n= 8; QC, n= 8, AraC+QC, n= 8; ALL10, ALL11: n= 8 or all groups). (B) QC delays ALL relapses and improves survival of primary recipients. Survival of recipients transplanted with ALL9 (Left), ALL10 (Middle) or ALL11 (Right) cells were monitored and plotted by Kaplan-Meier method (ALL9: Ctr, n= 10; V, n= 9; AraC, n= 8; QC, n= 9, AraC+QC, n= 9; ALL10: Ctr, n= 10; V, n= 9; AraC, n= 8; QC, n= 12, AraC+QC, n= 12; ALL11: Ctr, n= 10; V, n= 9; AraC, n= 8; QC, n= 12, AraC+QC, n= 12). Median survival of ALL9 transplant: Vehicle (35 days); AraC (55 days); QC (45 days) and AraC+QC (70 days). Median survival of ALL10 transplant: Vehicle (34 days); AraC (55 days); QC (46 days) and AraC+QC (62 days). Median survival of ALL11 transplant: Vehicle (33 days); AraC (55 days); QC (48 days) and AraC+QC (73 days). (C) QC fails to improve survival of secondary transplanted recipients. hCD45$^+$ cells from the primary recipients (B) were sorted and pooled, then transplanted into sublethally irradiated NSGS mice. Survival of recipients transplanted with ALL9 (Left), ALL10 (Middle) or ALL11 (Right) cells were monitored and plotted by Kaplan-Meier method (ALL9: Ctr, n= 10; V, n= 9; AraC, n= 10; QC, n= 9, AraC+QC, n= 8; ALL10: Ctr, n= 10; V, n= 9; AraC, n= 10; QC, n= 9, AraC+QC, n= 9; ALL11: Ctr, n= 10; V, n= 9; AraC, n= 10; QC, n= 9, AraC+QC, n= 9). V, Vehicle; A, AraC; QC: Quinacrine; A+QC, AraC+Quinacrine. Median survival of ALL9 transplant: Vehicle (23 days); AraC (50 days); QC (35 days) and AraC+QC (57.5 days). Median survival of ALL10 transplant: Vehicle (27 days); AraC (52 days); QC (35 days) and AraC+QC (55 days). Median survival of ALL11 transplant: Vehicle (24 days); AraC (51 days); QC (35 days) and AraC+QC (59 days). Statistics were performed in the indicated groups: two-sided paired *t*-test (parametric). Animal survival data were analyzed

by Gehan-Breslow-Wilcoxon test; p values are indicated in Source Data files (*p<0.05; ** p<0.01; *** p<0.001; **** p<0.0001).

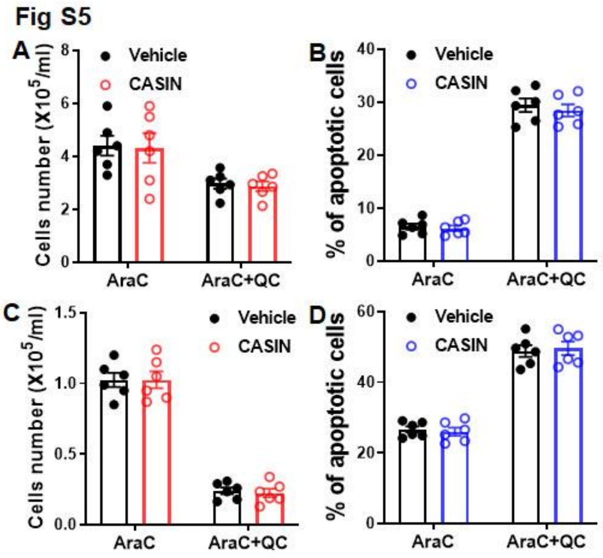

Supplementary Figure 5. CASIN does not enhance the cytotoxicity of AraC+QC regimen on ALL cells *in vitro*. (A, B) AraC-resistant Molt4-Luc2 cells were cultured in the presence of AraC (2 μM) and QC (100 nM) in the presence or absence of CASIN (5 μM) for 48 hours. Viable cells were enumerated (A) or subjected to flow cytometry analysis for Annexin V and 7AAD (B). All data represent are mean ± SD of three independent experiments (n= 6). (C, D) Primary ALL cells were cultured on hTERT-immortalized MSCs, and treated for one week with AraC (500 nM) and QC (100 nM) in the presence or absence of CASIN (5 μM). Viable cells were enumerated (C) or subjected to flow cytometry analysis for Annexin V and 7AAD (D). (n= 6) Statistics were performed in the indicated groups: Two-tailed, paired *t* test (parametric); p values are indicated in Source Data files.

Fig S6

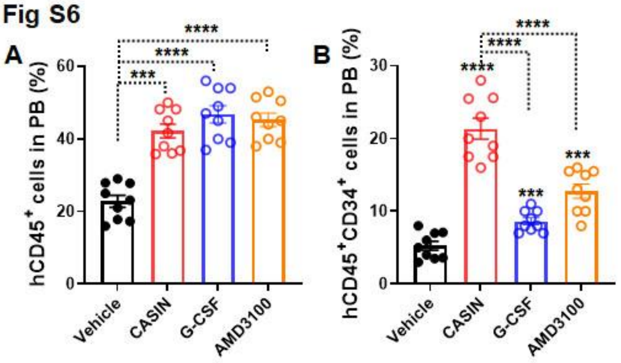

Supplementary Figure 6. CASIN is superior in mobilizing LSC-enriched ALL cells than G-CSF and AMD3100. NSGS mice transplanted with primary patient ALL cells were administrated with CASIN (2.5 mg/kg, IP), G-CSF (100 mg/kg per day once a day for 5 days, IP) or AMD3100 (5 mg/kg, IP). Percentages of hCD45^{+} cells (A) and hCD45^{+}CD34^{+} cells (B) in the peripheral blood (PB) were determined by flow cytometry. Results are mean ± SEM of three independent experiments (n= 9/group). Statistics were performed in the indicated groups: Two-tailed, paired *t* test (parametric); p values are indicated in Source Data files (*** p<0.001; **** p<0.0001).

Fig S7

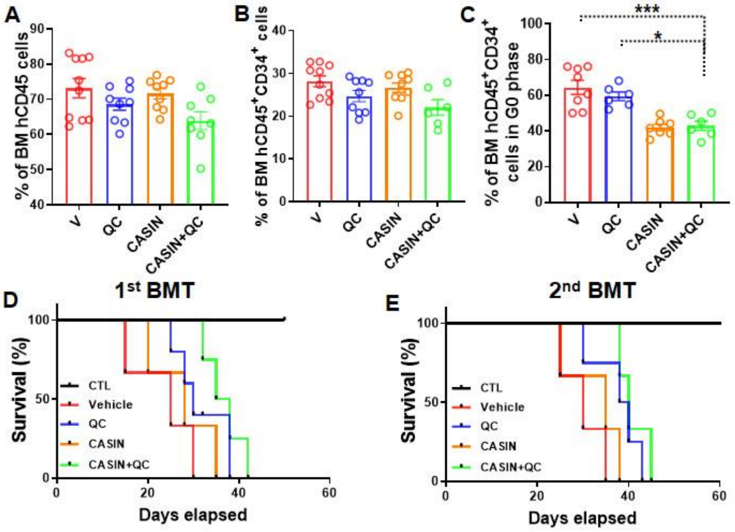

Supplementary Figure 7. CASIN enhances the eradication of ALL LSCs by AraC+QC and delays ALL relapse. (A) CASIN decreases total $hCD45^+$ cells in the BM of primary recipients. 1- 2×10^6 primary ALL cells were transplanted into sublethally irradiated NSGS mice followed by the treatments described in Fig 5A. BM cells from the recipients were subjected to flow cytometry analysis for hCD45 (V, n= 10; QC, n= 9; CASIN, n= 9; CAIN+QC, n= 8). (B) CASIN decreases LSC-enriched cell population in the BM of primary recipients. BM cells from recipients described in (A) were subjected to flow cytometry analysis for hCD45 and hCD34 (V, n= 10; QC, n= 9; CASIN, n= 9; CAIN+QC, n= 8). (C) CASIN reduces quiescent LSC-enriched ALL cells in the BM of the primary recipients. BM cells from recipients described in (A) were gated for cell cycle analysis of $hCD45^+$ $hCD34^+$ cells (V, n= 8; QC, n= 6; CASIN, n= 7; CAIN+QC, n= 6). (D, E) CASIN improves survival of both primary and secondary recipients. BM cells from recipients of the same donor described in (A) were pooled and transplanted into sublethally irradiated NSGS recipients. Survival of the primary recipients (D, Ctr, n= 10; V, n= 9; QC, n= 12; CASIN, n= 9; CASIN+QC, n= 8) and secondary recipients (E, Ctr, n= 10; V, n= 9; QC, n= 12; CASIN, n= 9; CASIN+QC, n= 9) were monitored and plotted by Kaplan-Meier method (). Mice without transplantation served as controls (CTL). V, Vehicle; A, AraC; QC: Quinacrine; C, CASIN; A+QC, AraC+Quinacrine; A+QC+C, CASIN+AraC+Quinacrine. Statistics were performed in the indicated groups: two-sided paired *t*-test; animal survival data were analyzed by Gehan-Breslow-Wilcoxon test; p values are indicated in Source Data files (** $p<0.01$; *** $p<0.001$).
