# Supplementary material for: Quinacrine-CASIN combination overcomes chemoresistance in human acute lymphoid leukemia
Source: Nat Commun. 2021 Nov 26;12:6936. doi: 10.1038/s41467-021-27300-w (PMC8626516; doi:10.1038/s41467-021-27300-w)
Supplement: Supplementary file 2 — Reporting summary [file 41467_2021_27300_MOESM2_ESM.pdf]

nature research

Corresponding author(s): Wei Du

Last updated by author(s): 11/8/2021

Reporting Summary

Nature Research wishes to improve the reproducibility of the work that we publish. This form provides structure for consistency and transparency in reporting. For further information on Nature Research policies, see our Editorial Policies and the Editorial Policy Checklist.

Statistics

For all statistical analyses, confirm that the following items are present in the figure legend, table legend, main text, or Methods section.

n/a	Confirmed	
☐	☒	The exact sample size (*n*) for each experimental group/condition, given as a discrete number and unit of measurement
☐	☒	A statement on whether measurements were taken from distinct samples or whether the same sample was measured repeatedly
☐	☒	The statistical test(s) used AND whether they are one- or two-sided<br>*Only common tests should be described solely by name; describe more complex techniques in the Methods section.*
☐	☒	A description of all covariates tested
☐	☒	A description of any assumptions or corrections, such as tests of normality and adjustment for multiple comparisons
☐	☒	A full description of the statistical parameters including central tendency (e.g. means) or other basic estimates (e.g. regression coefficient) AND variation (e.g. standard deviation) or associated estimates of uncertainty (e.g. confidence intervals)
☐	☒	For null hypothesis testing, the test statistic (e.g. *F*, *t*, *r*) with confidence intervals, effect sizes, degrees of freedom and *P* value noted<br>*Give P values as exact values whenever suitable.*
☒	☐	For Bayesian analysis, information on the choice of priors and Markov chain Monte Carlo settings
☒	☐	For hierarchical and complex designs, identification of the appropriate level for tests and full reporting of outcomes
☒	☐	Estimates of effect sizes (e.g. Cohen's *d*, Pearson's *r*), indicating how they were calculated

Our web collection on statistics for biologists contains articles on many of the points above.

Software and code

Policy information about availability of computer code

Data collection	Flow cytometry data were collected using FACSDiva software v 6.1.3 on a FCS LSRII or FACS LSRFortessa analyzer (BD Biosciences, San Jose, CA).<br>The distribution (luciferase intensity) of Molt4-Luc2 cells in the mice were determined by visualizing luciferase using Xenogen IVIS imaging system (PerkinElmer/CAliper Life Science, Waltham, MA).<br>Western blot images were taken using Amersham Imager 680 chemiluminescence imaging system for both colorimetric markers and chemiluminescence images (Amersham Biosciences, Pittsburgh, PA).
Data analysis	Flow cytometry data were analyzed using FCSExpress software 7.08.0018 (De Novo Software, Pasadena, CA).<br>For IVIS imaging, the photons emitted from Molt4-Luc2, expressed as Flux (photons/s/cm2/steradian), were quantified and analyzed using the “Living image” software (Caliper Life Science, Waltham, MA). Living Image analysis and acquisition software 4.7 (Caliper Life Sciences, Waltham, MA) was used for analysis.<br>GraphPad Prism 9.0 (San Diego, CA) was used to draw graphs and statistical analysis in the study.<br>Microsoft PowerPoint 2010 and Photoshop CS3 was used to crop images from unprocessed images.<br>Quantification of the obtained western blots was performed by densitometry on ImageJ 1.8.0 (Bethesda, MD)<br><br>All the other data are available in the main text or the supplementary materials. the raw data that support all the remaining figures/tables are provided in the Source Data File.

For manuscripts utilizing custom algorithms or software that are central to the research but not yet described in published literature, software must be made available to editors and reviewers. We strongly encourage code deposition in a community repository (e.g. GitHub). See the Nature Research guidelines for submitting code & software for further information.

Data

Policy information about availability of data

All manuscripts must include a data availability statement. This statement should provide the following information, where applicable:

- Accession codes, unique identifiers, or web links for publicly available datasets
- A list of figures that have associated raw data
- A description of any restrictions on data availability

All raw data that support all the remaining figures/tables are provided in the Source Data File.

Field-specific reporting

Please select the one below that is the best fit for your research. If you are not sure, read the appropriate sections before making your selection.

[x] Life sciences [ ] Behavioural & social sciences [ ] Ecological, evolutionary & environmental sciences

For a reference copy of the document with all sections, see nature.com/documents/nr-reporting-summary-flat.pdf

Life sciences study design

All studies must disclose on these points even when the disclosure is negative.

Sample size	No Sample size calculation was performed. Sample size was determined according to previous published literature.<br>References:<br>Himburg HA, 2016; Di Tullio A, 2017; Guo et al., 2014
Data exclusions	When applicable, positive and negative controls were used in each experiment.<br>If these controls failed, the whole set of data was excluded.
Replication	Replicate numbers for individual experiments were listed in figure legend and supplementary. Briefly, all NSGS experiments have at least 3 biological replicates, growth curve OD measurement have 4 technical replicates for each genotype. all attempts on replication were successful.<br>In general, all experiments were generated from 2-3 biological replicate samples. Biological replicate samples were generated independent of each other. All attempts on replication were successful.
Randomization	No randomization was performed. All strains used in this study are isogenic and are therefore considered identical.<br>Any genetic manipulation and chemical treatment is therefore intrinsically randomized.
Blinding	Investigators were not blinded as this was not relevant to the analysis of the data generated here, and the same pipelines and scripts were used to analyze all samples.

Reporting for specific materials, systems and methods

We require information from authors about some types of materials, experimental systems and methods used in many studies. Here, indicate whether each material, system or method listed is relevant to your study. If you are not sure if a list item applies to your research, read the appropriate section before selecting a response.

Materials & experimental systems

n/a	Involved in the study	
[ ]	[x]	Antibodies
[ ]	[x]	Eukaryotic cell lines
[ ]	[ ]	Palaeontology and archaeology
[ ]	[x]	Animals and other organisms
[ ]	[x]	Human research participants
[ ]	[ ]	Clinical data
[ ]	[ ]	Dual use research of concern

Methods

n/a	Involved in the study	
[ ]	[ ]	ChIP-seq
[ ]	[x]	Flow cytometry
[ ]	[ ]	MRI-based neuroimaging

Antibodies

Antibodies used	For analysis or sorting of ALL and HSPCs derived from human cord blood or adult BM cells, we used:<br>hCD45 microbeads (Miltenyi Biotec, Auburn CA).<br>hCD45-PE Cy7 (Clone: H30, Cat: 560915; BD Bioscience),<br>mCD45-PerCP Cy5.5 (Clone:30-F11, Cat: 561869, BD Bioscience),

Lineage-FITC (Lin1, Cat: 340546, BD Bioscience),
CD34-PE (Clone 581, Cat: 560941, dilution: 1 in 25, BD Bioscience),
CD38-APC (Clone HIT2, Cat: 555462, BD Bioscience)
Human grafts in mice were assessed using:
CD19-FITC (Clone: H1B19; Cat: 555412, BD Bioscience),
CD3-APC (BD Bioscience, Clone: UCHT1, Cat: 561811),
hCD45-PeCy7 (BD Bioscience, Clone: H30, Cat: 560915), and
mCD45PerCPCy5.5 (Clone:30-F11, Cat: 550994) (BD Biosciences, San Jose, CA).
Annexin V and 7-AAD staining, or cell cycling analysis using Ki67 and DAPI staining.
all listed above were from BD Bioscience, San Jose, CA.

antibodies used for western blotting include:
LC3A/B (#4108S, Cell Signaling technology)
b-actin (Clone AC-74, Cat # A2228, sigma-Aldrich)
p65 (#8242S; Cell Signaling technology)
p62 (#5114, Cell Signaling technology)
phosph-p65 (#3033, Clone 93H1, Cell Signaling technology)

Validation

All antibodies used have been validated by the manufacturer and used according to the manufacturer's instructions. the manufacturer's websites staining the validation of each antibody are listed above. antibodies have been re-validated by titrating their concentration.
Optimal antibody concentration was used, depicted in the method section of the manuscript.
Isotypes and FMO were included in the experiments whenever appropriate.

Eukaryotic cell lines

Policy information about cell lines

Cell line source(s)

Molt4-Luc 2 cells and hTERT-immortalized primary bone marrow mesenchymal stroma cells (MSCs) were obtained from ATCC. Generation of AraC resistant Molt4-Luc2 cell lines are described in Supplementary materials.

Authentication

Cells were authenticated by examination of morphology and growth characteristics.

Mycoplasma contamination

Mycoplasma contamination was checked annually. Results are negative.

Commonly misidentified lines (See ICLAC register)

No commonly misidentified cell lines were utilized in the study.

Palaeontology and Archaeology

Specimen provenance

N/A

Specimen deposition

N/A

Dating methods

N/A

☒ Tick this box to confirm that the raw and calibrated dates are available in the paper or in Supplementary Information.

Ethics oversight

N/A

Note that full information on the approval of the study protocol must also be provided in the manuscript.

Animals and other organisms

Policy information about studies involving animals; ARRIVE guidelines recommended for reporting animal research

Laboratory animals

NSGS mice were obtained from Jackson Laboratory and bred in equipped animal facility at University of pittsburgh. The 8-10 week old mice (both male and female) were used for experiments.
By default, animal housing areas are on a 12 hour x 12 hour light/dark cycle without twilight with most 7 AM on and 7 PM off and adjusting to remain synchronized with Eastern Standard Time (EST) and Daylight Saving Time (DST).
Temperatures of 65-75°F (~18-23°C) with 40-60% humidity are maintained.

Wild animals

no wild animals used in the study

Field-collected samples

no field collected samples used in the study

Ethics oversight

All animal experiments were carried out in accordance with the National Institutes of Health Guidelines for the Care and Use of Laboratory Animals and approved by the Institutional Animal Care and Use Committee of University of Pittsburgh.

Note that full information on the approval of the study protocol must also be provided in the manuscript.

Human research participants

Policy information about studies involving human research participants

Population characteristics	Patient samples were obtained randomly from healthy donors and patients with ALL at diagnosis. Patient characterization is depicted in the supplementary table S2.
Recruitment	Primary samples with the proper clinical diagnosis were collected after informed consent was obtained from all patients. The selection of patients was random and we believe there was no bias in patients sample collection
Ethics oversight	Primary human ALL cells were obtained after informed consent at West Virginia University Cancer Center; Cincinnati Children's Hospital Medical Center Respiration Core; and Pittsburgh Biospecimen Core under the approved Institutional Review Broad (IRB) protocols: #1310105737; STUDY19030357 and # 2011-3023.

Note that full information on the approval of the study protocol must also be provided in the manuscript.

Clinical data

Policy information about clinical studies

All manuscripts should comply with the ICMJEguidelines for publication of clinical research and a completedCONSORT checklist must be included with all submissions.

Clinical trial registration	N/A
Study protocol	N/A
Data collection	N/A
Outcomes	N/A

Dual use research of concern

Policy information about dual use research of concern

Hazards

Could the accidental, deliberate or reckless misuse of agents or technologies generated in the work, or the application of information presented in the manuscript, pose a threat to:

No	Yes	
☒	☐	Public health
☒	☐	National security
☒	☐	Crops and/or livestock
☒	☐	Ecosystems
☒	☐	Any other significant area

Experiments of concern

Does the work involve any of these experiments of concern:

No	Yes	
☒	☐	Demonstrate how to render a vaccine ineffective
☒	☐	Confer resistance to therapeutically useful antibiotics or antiviral agents
☒	☐	Enhance the virulence of a pathogen or render a nonpathogen virulent
☒	☐	Increase transmissibility of a pathogen
☒	☐	Alter the host range of a pathogen
☒	☐	Enable evasion of diagnostic/detection modalities
☒	☐	Enable the weaponization of a biological agent or toxin
☒	☐	Any other potentially harmful combination of experiments and agents

ChIP-seq

Data deposition

- [ ] Confirm that both raw and final processed data have been deposited in a public database such as GEO.
- [ ] Confirm that you have deposited or provided access to graph files (e.g. BED files) for the called peaks.

Data access links<br>*May remain private before publication.*	N/A
Files in database submission	N/A
Genome browser session<br>(e.g. UCSC)	N/A

Methodology

Replicates	N/A
Sequencing depth	N/A
Antibodies	N/A
Peak calling parameters	N/A
Data quality	N/A
Software	N/A

Flow Cytometry

Plots

Confirm that:

- [x] The axis labels state the marker and fluorochrome used (e.g. CD4-FITC).
- [x] The axis scales are clearly visible. Include numbers along axes only for bottom left plot of group (a 'group' is an analysis of identical markers).
- [x] All plots are contour plots with outliers or pseudocolor plots.
- [x] A numerical value for number of cells or percentage (with statistics) is provided.

Methodology

Sample preparation

For analysis and sorting of ALL and HSPCs derived from human Cord Blood or adult BM, cells were stained with hCD45-PE Cy7 (Clone: H30, Cat: 560915), mCD45-PerCP Cy5.5 (Clone:30-F11, Cat: 550994), Lineage-FITC (lin1, Cat: 340546), CD34-PE (Clone 581, Cat: 560941, dilution: 1 in 25), and CD38-APC (Clone HIT2, Cat: 555462) (all from BD Biosciences, San Jose, CA). Human grafts in mice were assessed using CD19-FITC (Clone: H1B19; Cat: 555412), CD3-APC (Clone: UCHT1, Cat: 561811), hCD45-PeCy7 (Clone: H30, Cat: 560915), and mCD45PerCPCy5.5 (Clone:30-F11, Cat: 550994) (all from BD Biosciences, San Jose, CA). Non-viable cells were excluded by DAPI staining. Appropriate isotype-matched antibodies were used as controls. Flow cytometry analysis was performed using an LSRII flow cytometer (BD Biosciences, San Jose, CA). Cell sorting was performed using a FACS Aria or INFLUX (BD Biosciences, San Jose, CA). FACSDiva software v 6.1.3 was used for data acquisition (BD Biosciences, San Jose, CA).
Cells were subjected to the indicated mice followed by flow cytometry analysis to determine cell apoptotic status following Annexin V and 7-AAD staining, or cell cycling analysis using Ki67 and DAPI staining.
For BrdU incorporation assay, Bromodeoxyuridine (BrdU, 150 ul of 10 mg/ml) were intraperitoneally (i.p.) injected to subjected mice followed by BM cells isolation 14 hours later. BrdU incorporated cells (S phase) were analyzed with the APC BrdU Flow Kit (BD Biosciences, San Jose, CA), following the manufacturer's instructions. Briefly, cells were surface stained then fixed and permeabilized using BD Cytofix/Cytoperm Buffer. After 1 hour incubation with DNase at 37 °C, cells were stained with APC-conjugated anti-BrdU monoclonal antibody. 7-aminoactinomycin (7-AAD) was added to each sample right before Flow Cytometry analysis (BD Biosciences, San Jose, CA).
For mitoSOX staining, treated cells were stained with MitoSOX (5 uM, Molecular Pribes, Waltham. MA) at 37 °C for 10 mi in the dark then washed with pre-warmed PBS. cell pellets were suspended in pre-warmed PBS followed by Flow cytometry analysis.
For intracellular phos-p65 staining, cells were fixed and permeabilized using BD Cytofix/Cytoperm Buffer and stained with pho-p65 antibody (#3033S, Clone 93H1, Cell Signaling Tech, Beverly, MA) for 30 min followed by secondary Antibody incubation. PBS washed cells were then subjected to flow cytometry analysis.

Instrument

LSRFortessa Flow cytometer (BD Bioscience, USA)
Aria II FACS (BD) for FACS sorting

Software	Flow cytometry data were collected using FACSDiva software v 6.1.3 on a FCS LSRII or FACS LSRFortessa analyzer (BD Biosciences, San Jose, CA).<br>FCSExpress software 7.08.0018 (De Novo Software, Pasadena, CA) was used for data analysis.
Cell population abundance	human CD45 cell purity after hCD45 microbeads positive selection was more than 95%.
Gating strategy	Each relevant gating strategy was mentioned in the figure legends or showed in the figures. prior to gating cell population o interest, cell debris was removed based on FCS/SSC and only live cell population were analyzed.

☒ Tick this box to confirm that a figure exemplifying the gating strategy is provided in the Supplementary Information.

Magnetic resonance imaging

Experimental design

Design type	N/A
Design specifications	N/A
Behavioral performance measures	N/A

Acquisition

Imaging type(s)	N/A
Field strength	N/A
Sequence & imaging parameters	N/A
Area of acquisition	N/A

Diffusion MRI ☐ Used ☒ Not used

Preprocessing

Preprocessing software	Microsoft PowerPoint 2010 and Photoshop CS3 (V23.0) was used to crop images from unprocessed images.
Normalization	N/A
Normalization template	N/A
Noise and artifact removal	N/A
Volume censoring	N/A

Statistical modeling & inference

Model type and settings	Graphpad Prism 9.0 (San Diego, CA) was used for all statistical analysis. Paired or unpaired student's t-test was used for two-group comparisons. Survival data were plotted by the Kaplan-Meier curve method and analyzed by the Gehan-Breslow-
Effect(s) tested	N/A

Specify type of analysis: ☐ Whole brain ☐ ROI-based ☐ Both

Statistic type for inference<br>(See Eklund et al. 2016)	N/A
Correction	N/A

Models & analysis

n/a	Involved in the study
☒	☐ Functional and/or effective connectivity
☒	☐ Graph analysis
☒	☐ Multivariate modeling or predictive analysis
